# Elevated glucocorticoid alters the developmental dynamics of hypothalamic neurogenesis in zebrafish

**DOI:** 10.1038/s42003-024-06060-5

**Published:** 2024-04-05

**Authors:** Helen Eachus, Min-Kyeung Choi, Anna Tochwin, Johanna Kaspareit, May Ho, Soojin Ryu

**Affiliations:** 1 Living Systems Institute & Department of Clinical and Biomedical Sciences, University of Exeter, Stocker Road, Exeter, EX4 4QD UK; 2 Institute of Human Genetics, University Medical Center, Johannes Gutenberg University Mainz, Langenbeckstraße 1, 55131 Mainz, Germany; 3 Nanyang Technological University, 50 Nanyang Avenue, Singapore, 639798 Singapore; 4 Present Address: Institute of Health and Neurodevelopment & Aston Pharmacy School, Aston University, Birmingham, B4 7ET UK

**Keywords:** Neuroscience, Developmental biology

## Abstract

Exposure to excess glucocorticoid (GC) during early development is implicated in adult dysfunctions. Reduced adult hippocampal neurogenesis is a well-known consequence of exposure to early life stress or elevated GC, however the effects on neurogenesis during development and effects on other brain regions are not well understood. Using an optogenetic zebrafish model, here we analyse the effects of GC exposure on neurogenesis during development in the whole brain. We identify that the hypothalamus is a highly GC-sensitive region where elevated GC causes precocious development. This is followed by failed maturation and early decline accompanied by impaired feeding, growth, and survival. In GC-exposed animals, the developmental trajectory of hypothalamic progenitor cells is strikingly altered, potentially mediated by direct regulation of transcription factors such as *rx3* by GC. Our data provide cellular and molecular level insight into GC-induced alteration of the hypothalamic developmental trajectory, a process crucial for health across the life-course.

## Abstract (web summary)

A study in zebrafish indicates that elevated cortisol levels during early life can alter the developmental trajectory of the hypothalamus, inducing short-term precocious development, but leading to failed maturation in the long-term.

## Introduction

Glucocorticoids (GC) are the key effectors of the stress response and have pleiotropic effects on the body, acting to restore homoeostasis and thus allow an animal to respond adaptively to threat^1^. Since the developing brain is plastic, GC exposure during early life has the potential to alter the developmental trajectory of the brain. Indeed, alteration of the developmental trajectory may be an adaptive mechanism employed by animals exposed to stress during early life. For example, antenatal GC treatment has well documented effects on lung maturation, as well as other organs, in preterm babies^2^. However, such treatment has also been associated with negative outcomes in later life, such as increased incidence of mental health issues during childhood^3^, impacted development of fronto-parietal brain functions during adolescence^4^, and cortical thinning in children with associated affective disorders^5^. Early life GC exposure leading to adverse consequences in later life is an example of the concept known as early life programming of adult disease^6–9^.

One of the well-documented effects of GC exposure during early life or early life stress (ELS) is reduced adult neurogenesis in the hippocampus, including reduced cell proliferation^10–14^. Indeed, adrenalectomy is sufficient to prevent reduced proliferation in chronically stressed mice, suggesting that GC is the main driving force behind this phenotype^15^. In contrast, only a few studies have looked at the effects of ELS or GC exposure on neurogenesis during development, but there are some indications that its effects might be developmentally dynamic. Specifically, it is possible that GC-induced reduction of cell proliferation is a delayed consequence of a GC-induced change to the developmental trajectory that began in early life. Indeed, rats exposed to ELS exhibited enhanced hippocampal cell proliferation and improved stress-associated behavioural performance as young adults, whilst these effects were reversed by middle age^10^. A potential mechanistic explanation for the ELS-induced reduction in adult hippocampal neurogenesis is that enhanced cell proliferation at an earlier time-point depletes the stem cell pool over time^16^.

Despite the well-documented effects of GC exposure on hippocampal neurogenesis, the effects of GC exposure on other brain regions are unclear. A recent study revealed the effects of ELS on hypothalamic neurogenesis showing that cell proliferation and numbers of hypothalamic stem cells known as tanycytes were reduced in the adult mouse^17^. Tanycytes are unique radial glial-like cells that line the walls of the 3rd ventricle, have NSPC (neural stem/progenitor cell) properties that persist into adulthood, send projections into neighbouring hypothalamic nuclei and are able to sense glucose levels in the CSF^18^. Whilst most previous studies have focused on effects of stress and GC exposure on neurogenesis in the hippocampus, the aforementioned study supports that the hippocampus is not uniquely affected by stress/GC. However, whether and how GC affects hypothalamic neurogenesis during development is largely unknown.

Here we used an optogenetic zebrafish model in which the endogenous cortisol level is elevated, to test the hypothesis that GC exposure alters the developmental trajectory of the brain. The model utilises optogenetic manipulation of steroidogenic interrenal cells to increase the endogenous GC level during development^19–21^. Whilst the mammalian adrenal gland, counterpart of the fish interrenal, is known to produce GCs, mineralocorticoids (MCs), and androgens^22^; MCs and androgens are not detectable in the larval zebrafish interrenal gland^23–26^. Therefore, the phenotypes observed in our model are primarily caused by GC and its downstream effectors, rather than MC or androgen over-exposure.

We found that GC exposure affected cell proliferation in a region-specific manner and that these effects were dynamic across the life-course. We found that the hypothalamus is a highly GC-sensitive brain region. In early life, we observed precocious development of the hypothalamus and hypothalamus-associated feeding behaviour in GC-exposed animals. This was followed by a rapid decline, indicated by failed hypothalamic maturation, including reduced cell proliferation and altered numbers of hypothalamic neuronal subtypes, and suppressed feeding and growth. Our data uncover a tissue-specific time-dependent plasticity of the hypothalamus in response to GC and provide cellular and molecular insights for GC-induced alteration of the hypothalamic developmental trajectory.

## Results

### Neurogenesis is altered in an optogenetic zebrafish model of elevated GC

**Fig. 1:**
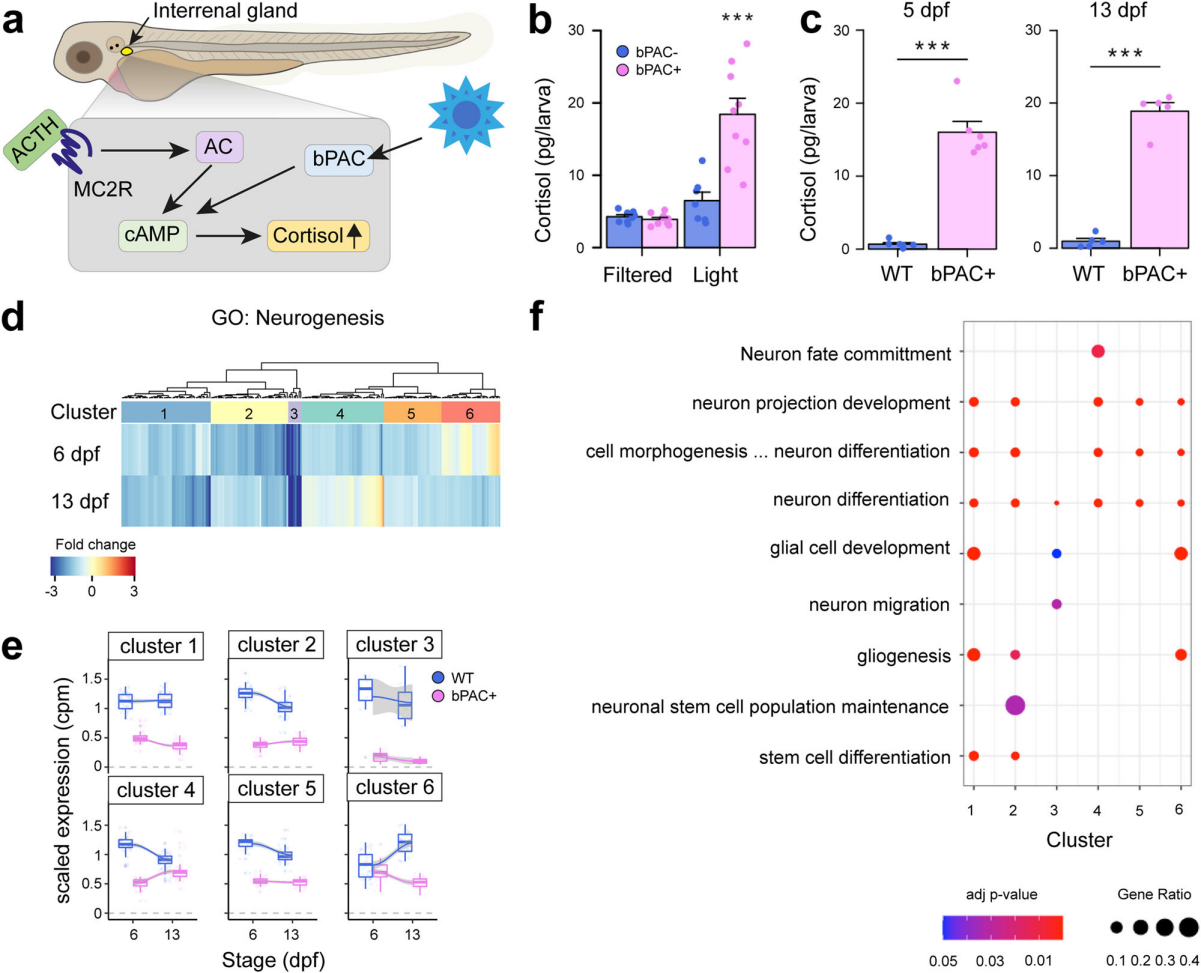
Neurogenesis is altered in an optogenetic zebrafish model of elevated GC. **a** In star:bPAC+ larvae, blue light activates cAMP signalling in the interrenal gland, which increases cortisol. Figure adapted from Eachus et al.^89^. **b** At 5 dpf, star:bPAC+ larvae exposed to white light for 10-min exhibit elevated cortisol levels compared to star:bPAC- siblings and compared to star:bPAC+ and star:bPAC- larvae raised under a blue light filter (two-way ANOVA followed by Tukey post-hoc test; genotype: treatment interaction, *F* = 21.16, d.f. = 1, 29; *P* = 0.000077). *N* = 8, 9 (filter) and 7, 9 (light) for star:bPAC- and star:bPAC+ respectively. ***p < 0.001 compared to other groups. **c** At 5 dpf and 13 dpf, star:bPAC+ larvae raised under standard aquarium lighting conditions exhibit elevated cortisol compared to wild types (WT) (5 dpf, *N* = 6 pools of 12 larvae per group, *t* = −10.354, df = 5.1774, *p* = 0.0001179; 13 dpf, *N* = 5 pools of 5 larvae per group, *t* = −14.403, df = 4.8525, *p* = 0.000036). **d** Heatmap showing fold change for 225 genes associated with GO:0022008 (neurogenesis) in star:bPAC+ whole-brain samples that were downregulated at either 6 dpf or 13 dpf (or both), relative to wild type, from an RNA-sequencing analysis. **e** Scaled expression (cpm) of neurogenesis-associated genes in star:bPAC+ and wild-type whole-brain samples at 6 dpf and 13 dpf, according to the 6 clusters shown in (**d**). Boxes show the median and quartiles, whiskers are inter-quartile range and grey shading shows the smoothed conditional means. **f** Significant slimmed neurogenesis child GO terms at *p* < 0.05, identified using g:Profiler are shown for each cluster. Size of the dot indicates the ratio of differentially expressed genes from each GO term, whilst the colour shows the adjusted *p* value.

We previously developed an optogenetic zebrafish line to induce elevated endogenous cortisol levels through *beggiatoa* Photoactivated Adenylate Cyclase (bPAC) activation in steroidogenic interrenal cells via blue light^19,20^. For the present study, we used the same construct to generate a new transgenic line, *Tg(star:bPAC-2A-tdTomato)*^*uex300*^, in a different strain, to obtain a higher cortisol level than previously feasible (Fig. 1a). After 10 min of exposure to ambient white light (containing a blue light component), 5 days post fertilisation (dpf) *Tg(star:bPAC-2A-tdTomato)*^*uex300*^ larvae exhibited elevated cortisol levels compared to, their negative siblings which lack the transgene (Fig. 1b). Notably, *Tg(star:bPAC-2A-tdTomato)*^*uex300*^ larvae shielded from blue light did not exhibit cortisol elevation, confirming that bPAC activation by blue light is responsible for cortisol elevation (Fig. 1b). To assess the impact of elevated GC on brain development, we raised transgenic fish under standard aquarium lighting, ensuring bPAC activation. This resulted in significantly elevated cortisol levels in *Tg(star:bPAC-2A-tdTomato)*^*uex300*^ compared to wild types during larval stages (5 dpf and 13 dpf, Fig. 1c)^21^. *Tg(star:bPAC-2A-tdTomato)*^*uex300*^ raised under standard light conditions are denoted throughout as star:bPAC+ or bPAC + .

To investigate the effects of elevated GC on brain development we analyzed bulk RNA-sequencing (RNA-seq) data of whole brain samples from star:bPAC+ and wild-type fish at different developmental stages^21^. Gene Ontology (GO) analysis of differentially expressed genes (DEGs) that were down-regulated in star:bPAC+ brains compared with wild types were enriched for a number of biological processes, including Neurogenesis at all developmental stages (GO:0022008, adjusted *p* value 2.47E-20 at 6 dpf, 5.45E-08 at 13 dpf, 1.11E-17 at 120 dpf). Up-regulated genes were not enriched for GO term neurogenesis. To investigate the effects of GC exposure on neurogenesis during development, we focused downstream analysis on the early life time-points: 5/6 dpf and 13 dpf. 225 genes associated with neurogenesis were down-regulated in star:bPAC+ brains at either 6 dpf or 13 dpf (Fig. 1d). To confirm our results and identify specific features of neurogenesis that are altered by elevated GC, we performed qPCR on independent samples of whole brains from 5 dpf star:bPAC+ and wild types for genes associated with neurogenesis processes or distinct cell types. Of the 14 categories of genes analysed, only one category was associated with consistent differential expression in star:bPAC+. Expression of *Pcna*, *mki67* and *mcm5*, all associated with cell proliferation, showed increased expression in star:bPAC+ compared with wild types (Supplementary Fig. 1). These data support that neurogenesis, and especially cell proliferation, is altered by elevated GC.

To identify whether the effect of GC on neurogenesis is developmentally dynamic, we performed unsupervised clustering analysis of neurogenesis DEGs at 6 dpf and 13 dpf and identified 6 different clusters. Cluster 2 and cluster 5 genes showed a relatively stronger down-regulation at 6 dpf compared to 13 dpf, meanwhile cluster 1 genes showed a relatively stronger downregulation at 13 dpf (Fig. 1e). Cluster 3 genes were strongly downregulated at both time-points (Fig. 1e). Interestingly, cluster 4 and cluster 6 genes showed a time point-specific down-regulation. Cluster 4 genes were downregulated in star:bPAC+ at 6 dpf but were not differentially expressed at 13 dpf (Fig. 1e). Meanwhile cluster 6 genes were down-regulated in star:bPAC+ at 13 dpf but not at 6 dpf (Fig.1e). These results suggest that some effects of elevated GC on neurogenesis are temporarily dynamic during early life. GO analysis of genes within the clusters indicated that all clusters contained genes associated with neuron differentiation (Fig. 1f). Gliogenesis and glial cell development were enriched in multiple clusters. There were also some terms which were restricted to single or small numbers of clusters. For example, cluster 4 genes were associated with neuron fate commitment, cluster 3 with neuron migration, and cluster 2 with neuronal stem cell population maintenance (Fig. 1f). These results suggest that specific aspects of neurogenesis exhibit temporally dynamic differential expression during early life as a result of GC exposure.

### Precocious hypothalamic development following elevated GC

**Fig. 2:**
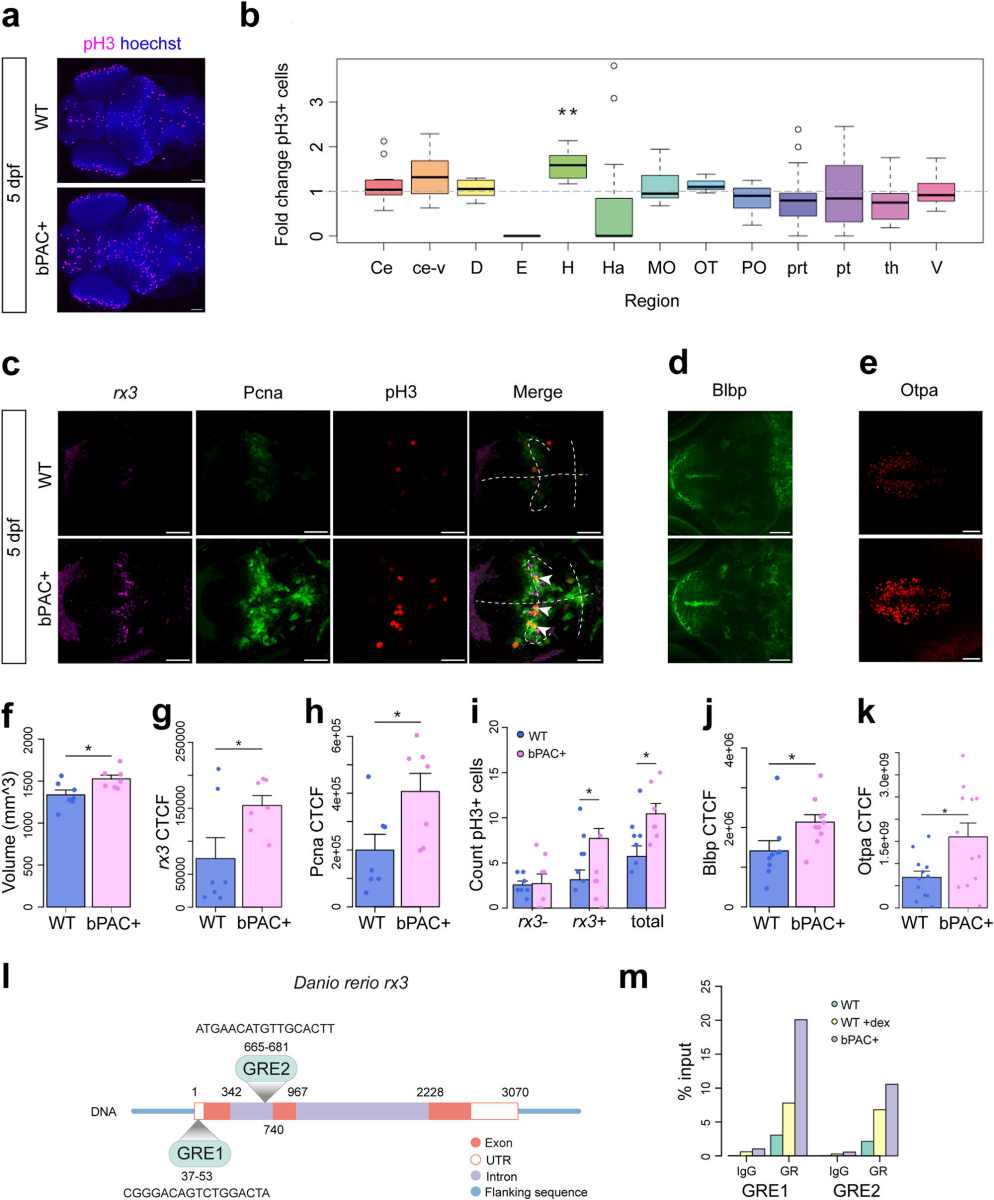
Precocious hypothalamic development following elevated GC. **a** Whole-brain IHC for proliferation marker pH3 as seen in a maximum intensity projection in 5 dpf wild-type and star:bPAC+ larvae. Scale bar is 60 μm. **b** Cell counting of pH3 across brain regions revealed an increase in the hypothalamus (H) of 5 dpf star:bPAC+ larvae, compared with wild type. Wilcox Exact test with Bonferroni correction (*N* = 14, *p* = 0.0013195). Brain regions are labelled according to the ZFIN anatomy database. The box shows the median next to the first and third quartile, whilst the whiskers indicate the first and third quartile ±1.5 interquartile range. **c** Co-expression of *rx3*, Pcna and pH3 in the hypothalamus of 5 dpf wild-type and star:bPAC+ larvae, observed in a single plane image of FISH-IHC. Scale bar 40 μm. Dashed line indicates the ventricles and arrowheads indicate excess *rx3* + pH3+ cells in star:bPAC+ hypothalamus. **d** IHC for Blbp in the arcuate nucleus of 5 dpf wild-type and star:bPAC+ larvae, observed in a single plane image. Scale bar 40 μm. **e** IHC for Otpa in the hypothalamus of 5 dpf wild-type and star:bPAC+ larvae, as observed in a maximum intensity projection. Scale bar 40 μm. The hypothalamus of 5 dpf star:bPAC+ larvae is larger (**f**, *t* = −2.6193, df = 11.207, *p* = 0.02353) and has increased intensity of *rx3* (**g**, *N* = 7, *t* = −2.3011, df = 8.5909, *p* = 0.04823) and Pcna (**h**, *N* = 7, *t* = −2.4409, df = 11.766, *p* = 0.03147). **i** Excess proliferating pH3+ cells in the hypothalamus of 5 dpf star:bPAC+ larvae are *rx3*+ radial glia (*N* = 7, Two-way ANOVA with Tukey’s test: total pH3+ cells (*p* = 0.0293550), *rx3* + pH3+ cells (*p* = 0.0373125)). Intensity of Blbp (**j**, *N* = 9, *t* = −2.2734, df = 14.903, *p* = 0.03823) and Otpa (**k**, *N* = 12, *t* = −2.44, df = 16.227, *p* = 0.02652) was increased in the hypothalamus of 5 dpf star:bPAC+ larvae. **l** The zebrafish *rx3* gene contains two GREs, one in exon one and another in intron one. The region shown is ENSDARG00000052893, GRCz11:21:10755554:10759823:1 (including flanking sequence). **m** ChIP qPCR analysis of two GREs in the *rx3* gene shows enrichment for GR compared with IgG control in 5 dpf wild-type brains. Enrichment of GR was higher in wild types exposed to dexamethasone, and higher still in star:bPAC+ brains for both GREs.

To determine whether the effects of elevated GC on cell proliferation were brain-wide or region specific we performed immunohistochemistry (IHC) for mitosis marker phospho-histoneH3 (pH3) on brains of 5 dpf star:bPAC+ and wild-type larvae (Fig. 2a). Cell counting of proliferating pH3+ cells across brain regions indicated a trend towards an increase in number of pH3+ cells in the valvula cerebelli (ce-v) of the hindbrain, however, the only significant increase was restricted to the hypothalamus (Fig. 2b). We next sought to determine which cell types were associated with the increased proliferative capacity in the hypothalamus of 5 dpf star:bPAC + . To this end, we performed Fluorescent in situ hybridisation (FISH) for *rx3* (*retinal homeobox gene 3)*, combined with IHC for Pcna and pH3. *rx3* is a transcription factor known to be expressed in hypothalamic progenitor cells in zebrafish where it plays a critical role in hypothalamic development^27^, and its mammalian orthologue *Rax* is known to be expressed in hypothalamic radial glia^28^. Pcna and pH3 are markers for proliferating cells during S-phase and M-phase respectively. Pcna+ cells were situated around the 3rd ventricle of the hypothalamus, especially around the lateral recess, in both star:bPAC+ and wild-type larvae, with a small number of pH3+ cells scattered throughout this region (Fig. 2c). We distinguish the Pcna+ region around the hypothalamic ventricles as the ‘proliferative zone’ and observed co-localisation of *rx3* with Pcna and pH3 within the proliferative zone (Fig. 2c, white arrowheads).

Quantitative analysis of confocal stacks revealed that the hypothalamus was significantly larger (Fig. 2f), and we found a significant increase in *rx3* and Pcna signal intensity, and significantly more pH3+ cells in the hypothalamus of 5 dpf star:bPAC+ compared with wild types (Fig. 2g–i). To determine whether the observed increase in hypothalamic proliferation in the hypothalamus of 5 dpf star:bPAC+ larvae is a direct effect of developmental GC exposure, we treated star:bPAC+ larvae with either the GR antagonist RU-486 or MR antagonist Spironolactone from 2 dpf until 5 dpf. Treatment with RU-486 did not significantly affect hypothalamic volume or hypothalamic Pcna signal, however it was sufficient to significantly reduce the number of pH3+ mitotic cells in the hypothalamus (Supplementary Fig. 2). Meanwhile treatment of star:bPAC+ larvae with Spironolactone had no effect on hypothalamus size, Pcna signal or pH3 cell count (Supplementary Fig. 2). This suggests that GR signalling is a significant driver of the increased hypothalamic proliferation observed in star:bPAC+ larvae. Further, we observed that the excess hypothalamic pH3+ cells were *rx3* + , meanwhile *rx3*-/pH3+ cells were not different (Fig. 2i). These results suggest that excess proliferating hypothalamic cells in star:bPAC+ are *rx3*-expressing radial glia. Interestingly, we identified two Glucocorticoid Response Elements (GREs) within the zebrafish *rx3* gene (Fig. 2l). GRE1 is in the 5’ UTR, which is most likely a regulatory region. ChIP-qPCR analysis revealed enrichment of GR antibody (compared with IgG) at both GREs in larval brain samples, supporting that GR does indeed bind to regulatory regions of the *rx3* gene (Fig. 2m). Further, enrichment of GR was higher in dexamethasone-treated wild-type samples, compared with wild-type controls, and higher still in star:bPAC+ samples. We speculate that the increase in bPAC+ brains relative to dexamethasone-treated wild types is related to differences in dose, delivery, and rhythmicity of exposure. This experiment shows that GC exposure directly regulates GR-mediated control of *rx3* gene expression in the brain.

Whilst *rx3* appeared to be co-expressed with Pcna in proliferating radial glia, we also analysed Blbp (brain lipid binding protein, fabp7a) localisation, which is known to be expressed in predominantly quiescent radial glia in the zebrafish brain^29^. Consistent with this, we observed strong expression of Blbp in cells lining the third ventricle in the Pcna-negative rostral domains of the hypothalamus, whilst some Blbp signal was also observed in Pcna-positive cells of the lateral recess (Fig. 2d). In 5 dpf star:bPAC+ larvae, we observed increased Blbp signal, in the basal hypothalamus (Fig. 2d, j), suggesting that increased numbers of glial cells might include both proliferative and quiescent subtypes. Further, we observed increased signal for Otpa (orthopedia homeobox a) in the hypothalamus of 5 dpf star:bPAC+ larvae (Fig. 2e, k), which is predominantly expressed in early post-mitotic neuronal precursors^30^, suggesting that the excess GC-exposed hypothalamic proliferative radial glia do exit the cell cycle, leading to increased numbers of neuronal precursors.

To assess the impact of excess hypothalamic progenitor cells and neuronal precursors at 5 dpf, we examined differentiation into hypothalamic neurons. Hypothalamic expression of *agrp* (*agouti-related peptide*), *avp*, *hcrt*, *pmch* (*pro-melanin-concentrating hormone*), and *pomc* showed no difference between star:bPAC+ larvae and wild type (Supplementary Fig. 3). However, a decrease in hypothalamic *crhb* (*corticotropin releasing hormone b)* expression in star:bPAC+ larvae was observed, consistent with heightened negative feedback to *crh*+ neurons due to elevated GC (Supplementary Fig. 3). Additionally, an increase in *npy* + (*neuropeptide y*) cells in the rostro-lateral intermediate hypothalamus, along with elevated cluster 3 and cluster 6 dopaminergic (DA, th + ) neurons in the hypothalamus/PT (posterior tuberculum), was noted in star:bPAC+ larvae compared to wild type (Supplementary Fig. 3). These findings suggest that the excess progenitor cells and neuronal precursors in star:bPAC+ larvae at 5 dpf may promote the differentiation of specific neuronal subtypes in the developing hypothalamus. Together these data suggest that elevated GC drives precocious development of the hypothalamus in zebrafish larvae.

### Progenitor cells fail to differentiate in the hypothalamus of star:bPAC+ larvae

**Fig. 3:**
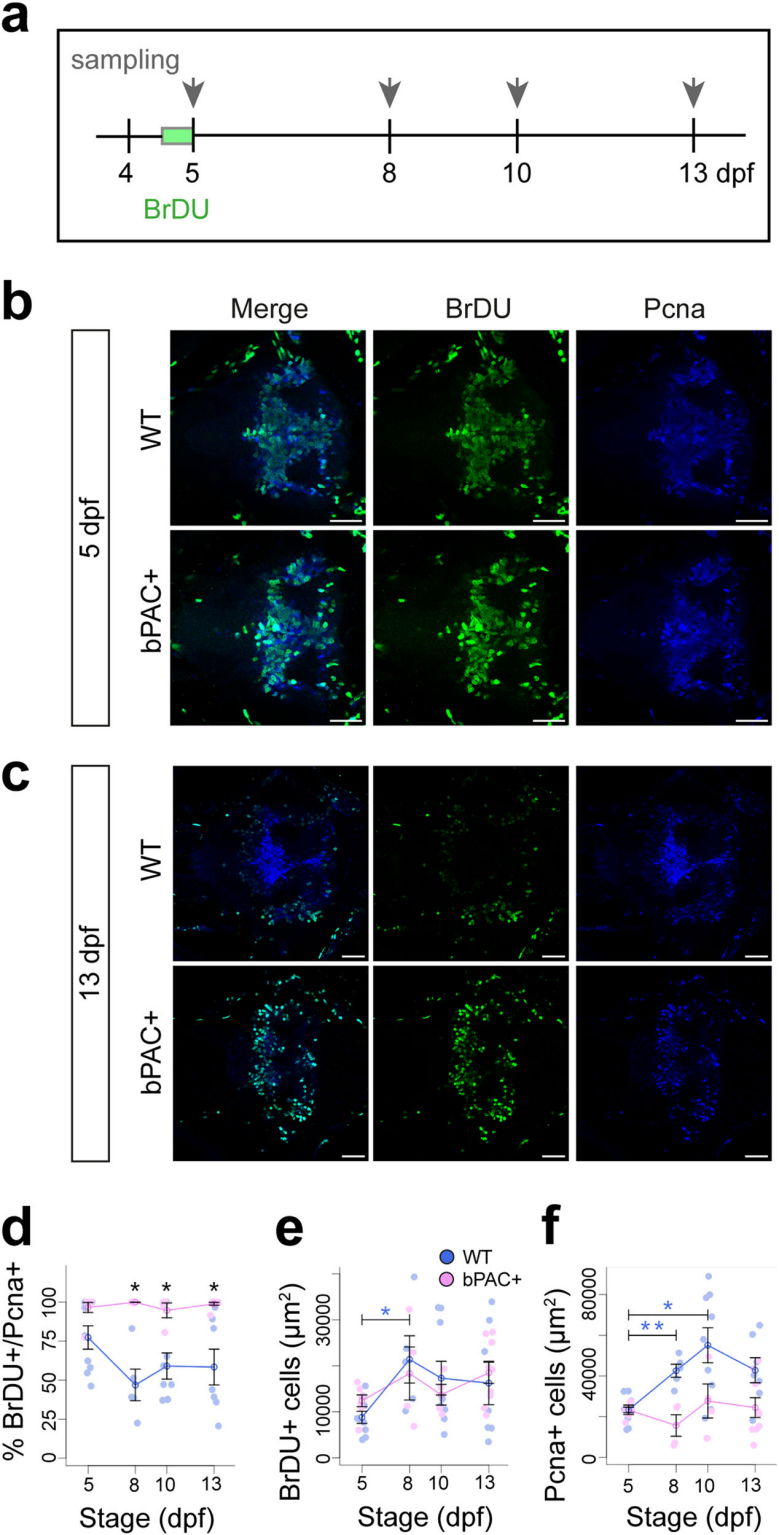
Progenitor cells fail to differentiate in the hypothalamus following elevated GC. **a** Schematic showing the experimental paradigm. Wild-type and star:bPAC+ larvae were treated with BrDU overnight from 4 dpf until 5 dpf for 17 h. Following removal of BrDU compound, larvae were collected at 5 dpf, 8 dpf, 10 dpf and 13 dpf for lineage tracing analysis. Confocal images of BrDU lineage tracing analysis at 5 dpf (**b**) and 13 dpf (**c**). In each panel wild types are shown in the upper row and star:bPAC+ larvae in the bottom row. The left-most column (Merge) shows the overlap between BrDU-labelled cells with endogenous Pcna, whilst single channel images for BrDU and Pcna are adjacent. All images are a single plane from a confocal z-stack. In all cases the scale bar indicates 40 μm. **d**−**f**. Quantitative analysis of BrDU+ cell fate. Whilst the percentage of BrDU+ cells expressing Pcna decreases over time in wild types, this remains high in star:bPAC+ (**d**). Whilst the area covered by BrDU+ cells increased between 5 dpf and 8 dpf in wild types, this does not occur in star:bPAC+ (**e**). Similarly, the area covered by Pcna+ cells increased between 5 dpf and 10 dpf in wild types, but not in star:bPAC+ larvae (**f**). Wild types are shown in blue, star:bPAC+ in pink. All graphs show the mean with standard error. 5 dpf *N* = 9, 7; 8 dpf *N* = 5, 4, 10 dpf *N* = 9, 4, 13 dpf *N* = 7, 9 wild type and bPAC+ respectively. *p < 0.05, **p < 0.01 for *t* tests with Bonferroni correction.

Since we observed excess proliferating radial glia in the hypothalamus of 5 dpf star:bPAC+ larvae, we sought to trace the proliferating cells of the hypothalamus after 5 dpf to determine their fate. To do this we exposed 4 dpf larvae to BrDU (Bromodeoxyuridine) overnight for 17 h until 5 dpf and then fixed larvae at 5, 8, 10 or 13 dpf for double-IHC analysis of BrDU and Pcna (Fig. 3a). At 5 dpf we observed successful incorporation of BrDU into proliferating cells, as observed by co-expression with Pcna, in the hypothalamus of both star:bPAC+ and wild-type larvae (Fig. 3b). By 8, 10 and 13 dpf, BrDU+ cells in the wild-type hypothalamus have begun exiting the cell cycle and are now mostly observed around the perimeter of the Pcna-expressing proliferative zone (Fig. 3c, Supplementary Fig. 4). Indeed, we observed that the percentage of hypothalamic BrDU+ cells that co-express Pcna drops from around 75% at 5 dpf to 50−55% at 8−13 dpf (Fig. 3d), suggesting that some of the BrDU+ cells that were proliferating at 5 dpf have lost their progenitor cell status and differentiated.

In stark contrast, hypothalamic BrDU+ cells in star:bPAC+ larvae are still predominantly observed within the Pcna+ proliferative zone after 5 dpf (Fig. 3c, Supplementary Fig. 4). In fact, the percentage of hypothalamic BrDU+ cells that co-express Pcna remains at around 100% from 5 dpf until 13 dpf in star:bPAC+ larvae (Fig. 3d). This indicates that in star:bPAC+ larvae, hypothalamic progenitor cells remain proliferative between 5 dpf and 13 dpf and do not differentiate. Further, we observed an increase in the number of hypothalamic BrDU+ cells between 5 dpf and 8 dpf in wild types, supporting that these cells are dividing and therefore increase in number, however no such increase was observed in star:bPAC+ larvae (Fig. 3e). Similarly, the number of hypothalamic Pcna+ cells increased in wild types between 5 dpf and 10 dpf, indicating that the proliferative zone continues to expand as cells divide during this period (Fig. 3f). No increase in the number of hypothalamic Pcna+ cells was observed in star:bPAC+ larvae during this developmental period (Fig. 3f), further supporting that neurogenesis appears to stall during this developmental window in star:bPAC+ larvae. Together, these data support that in star:bPAC+ larvae, hypothalamic progenitor cells fail to differentiate, and neurogenesis is stalled during the 5−13 dpf time window.

### Failed hypothalamic maturation following elevated GC

**Fig. 4:**
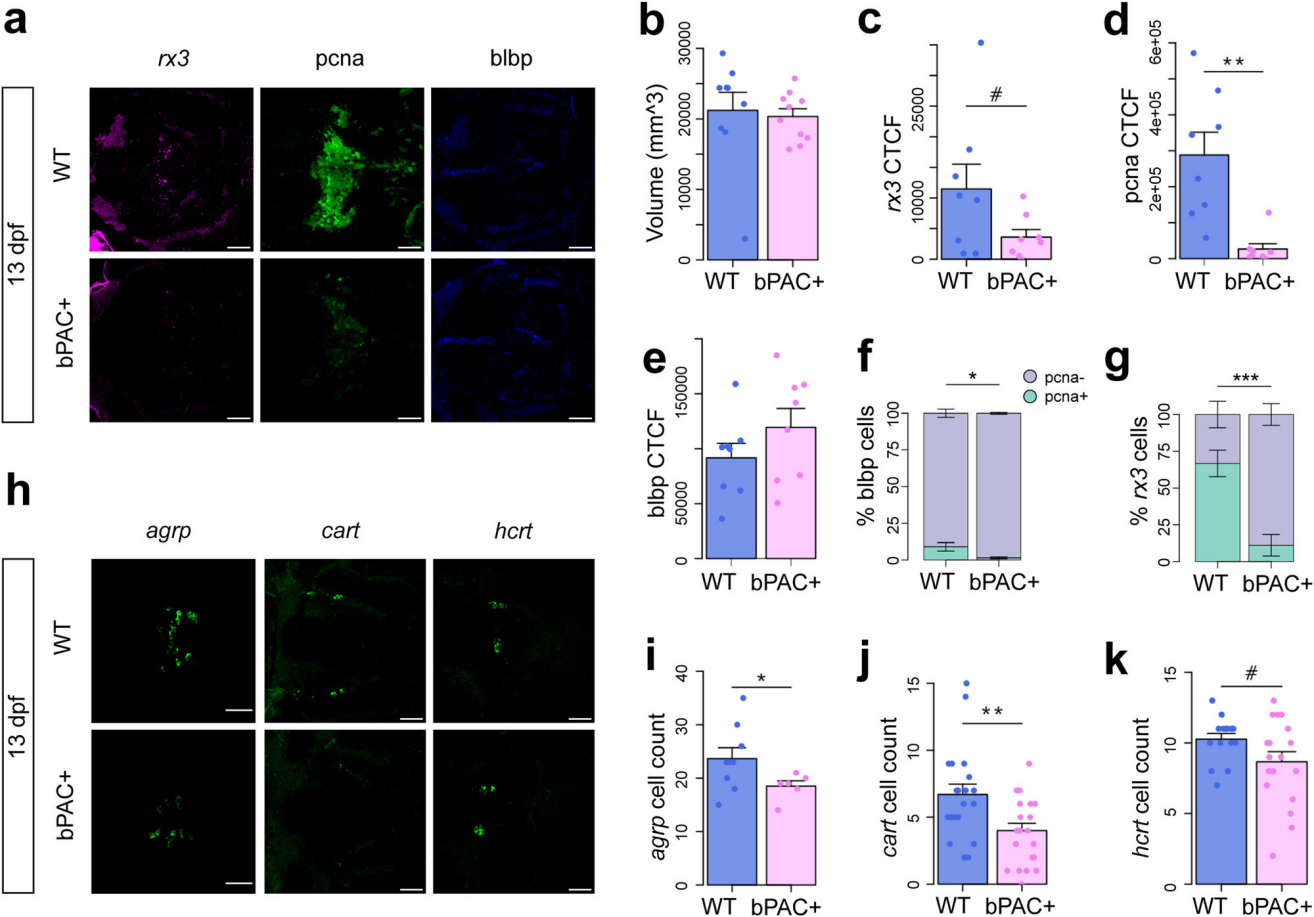
Failed hypothalamic maturation following elevated GC. **a** Expression of *rx3*, Pcna and Blbp in the hypothalamus of 13 dpf wild types and star:bPAC larvae, observed via FISH-IHC in single plane confocal images through the hypothalamus. Scale bar is 40 μm. **b-e** Quantitative analysis of confocal images shown in (**a**), *N* = 8. Volume of the hypothalamus is not different (**b**, *t* = 0.31771, df = 10.899, *p* = 0.7567), meanwhile, *rx3* CTCF shows a trend towards reduction (**c**, *t* = 1.8613, df = 8.3108, *p* = 0.09835), Pcna CTCF is significantly reduced (**d**, *t* = 4.0207, df = 7.7651, *p* = 0.00408), and Blbp CTCF is not significantly different (**e**, *t* = −1.2827, df = 13.092, *p* = 0.2219) in the hypothalamus of 13 dpf star:bPAC+ larvae compared with wild types. **f-g** Quantitative analysis of co-expression of Pcna in hypothalamic radial glia, from the images shown in (**a**). Blbp cells (**f**, *t* = −2.5888, df = 7.5126, *p* = 0.0339) and *rx3* cells (**g**, *t* = 4.7867, df = 13.444, *p* = 0.0003238) have a significant reduction in Pcna co-expression in the hypothalamus of 13 dpf star:bPAC+ larvae compared with wild types. **h** Expression of genes involved in feeding regulation in the hypothalamus of 13 dpf star:bPAC+ and wild-type larvae as shown in maximum intensity projections of confocal stacks. Scale bar is 40 μm. **i**−**k** Quantitative analysis of cell counts in the stacks shown in (**h**). Number of *agrp* cells is significantly lower (**i**, *N* = 9 wild type, *N* = 6 bPAC + , *t* = 2.2891, df = 11.255, *p* = 0.04234), number of *cart4* cells is significantly reduced (**j**, *N* = 20 wild type, *N* = 21 bPAC+ across 2 independent experiments, *t* = 2.8544, df = 34.862, *p* = 0.007212), number of *hcrt* cells shows a trend reduction (**k**, *N* = 15 wild type, *N* = 18 bPAC+ across 2 independent experiments, *t* = 1.9553, df = 26.571, *p* = 0.06115). All graphs show the mean with standard error. #*p* < 0.1, *p < 0.05, **p < 0.01, ***p < 0.001.

Since we observed that neurogenesis of proliferating hypothalamic cells was altered between 5 dpf and 13 dpf, we investigated hypothalamic cell populations at 13 dpf. In the 13 dpf hypothalamus, *rx3* was expressed in a subset of Pcna+ cells around the lateral recess, and Blbp was expressed mainly along the midline 3^rd^ ventricle anterior to the proliferative zone, in a similar manner to at 5 dpf (Fig. 4a). In 13 dpf star:bPAC+ larvae, we observed that the size of the hypothalamus was no longer larger than that of wild types, as we observed at 5 dpf (Fig. 4b). Expression of hypothalamic *rx3* was low at this developmental stage, and showed a trend towards a reduction in star:bPAC+ compared with wild type (Fig. 4a, c). In stark contrast to the 5 dpf hypothalamus, we now observed a dramatic reduction in expression of Pcna in 13 dpf star:bPAC+ hypothalamus (Fig. 4a, d). Meanwhile, Blbp signal intensity was not different between 13 dpf wild-type and star:bPAC+ larvae (Fig. 4a, e).

Strikingly, when we performed co-expression analysis, we observed that whilst only a small proportion of Blbp+ cells was proliferative in 13 dpf wild-type hypothalamus (~10%), almost all Blbp+ cells were quiescent in star:bPAC+ (Fig. 4f). Further, we observed that in wild types most hypothalamic *rx3+* cells (~70%) were proliferative and expressed Pcna, however in star:bPAC+ most *rx3+* cells (~85%) were quiescent and didn’t express Pcna (Fig. 4g). These data support that proliferative radial glia are lost in the hypothalamus of 13 dpf star:bPAC+ larvae. Next, we sought to identify an explanation for the loss of proliferative radial glia in the hypothalamus. We first analysed cell death in the hypothalamus of 13 dpf larvae using a TUNEL assay. We did not observe a significant difference in the number of apoptotic cells in the hypothalamus of 13 dpf star:bPAC+ larvae compared to wild types (Supplementary Fig. 5a), suggesting that loss of hypothalamic radial glia is not primarily due to increased cell death. We speculate that the loss of proliferation in the star:bPAC+ hypothalamus might be due to senescence of the formerly highly proliferative radial glia. In support of this hypothesis, we observed that one of the five significantly enriched KEGG pathways in our whole brain RNA-seq data set in the 13 dpf star:bPAC+ was cellular senescence, a pathway which was not enriched in the 6 dpf star:bPAC+ samples (Supplementary Fig. 5b, c). In the RNA-seq analysis, 102 of 150 genes associated with the KEGG pathway cellular senescence were differentially expressed in 13 dpf star:bPAC+ brain samples, meanwhile at 6 dpf only 60 of those genes were differentially expressed, and they generally showed a lesser degree of fold change (Supplementary Fig. 5d). This suggested that proliferative radial glia might have been lost in the hypothalamus of 13 dpf star:bPAC+ larvae due to cellular senescence, however further investigation of senescent markers in the hypothalamus is required to confirm this. Additionally, we confirmed that the loss of hypothalamic proliferation is maintained into later life, since we observed that in juvenile star:bPAC+ fish, the hypothalamus is smaller and contained fewer mitotic cells (Supplementary Fig. 6a–c). This suggests that the proliferative hypothalamic cells lost at 13 dpf do not re-enter the cell cycle later in development, suggesting that they have become senescent, rather than quiescent.

Since we observed a dramatic reduction in cell proliferation in the hypothalamus of 13 dpf star:bPAC+ larvae, we postulated that downstream neurogenesis and neuronal differentiation might also be affected. Firstly, analysis of Otpa+ neural precursor cells at 13 dpf revealed no difference between star:bPAC+ and wild types (Supplementary Fig. 7a, b). We next analysed hypothalamic neuronal subtypes in 13 dpf star:bPAC+ and wild type brains. *agrp*+ cells in the arcuate nucleus were significantly reduced in star:bPAC+ larvae (Fig. 4h, i). Similarly, *cart4* + (*cocaine- and amphetamine-regulated transcript-4*) cells in the rostro-lateral intermediate hypothalamus showed a decrease in star:bPAC+ larvae (Fig. 4h, j). Although a trend of reduction was observed in *hcrt*+ cells in the dorso-rostral hypothalamus of star:bPAC+ larvae (Fig. 4h, k), no differences were noted in *npy* + , *pomca* + , or *th*+ cells (Supplementary Fig. 7a, c, e, f). Notably, a significant increase in *pmch*+ cells was observed in the rostro-lateral intermediate hypothalamus of star:bPAC+ larvae compared to wild type (Supplementary Fig. 7a, d). These findings suggest that prolonged exposure to GC until 13 dpf affects neuronal differentiation and potentially disrupts the balance of hypothalamic neuronal subtypes in star:bPAC+ larvae.

### Functional consequences of elevated GC: precocious feeding followed by physical decline

**Fig. 5:**
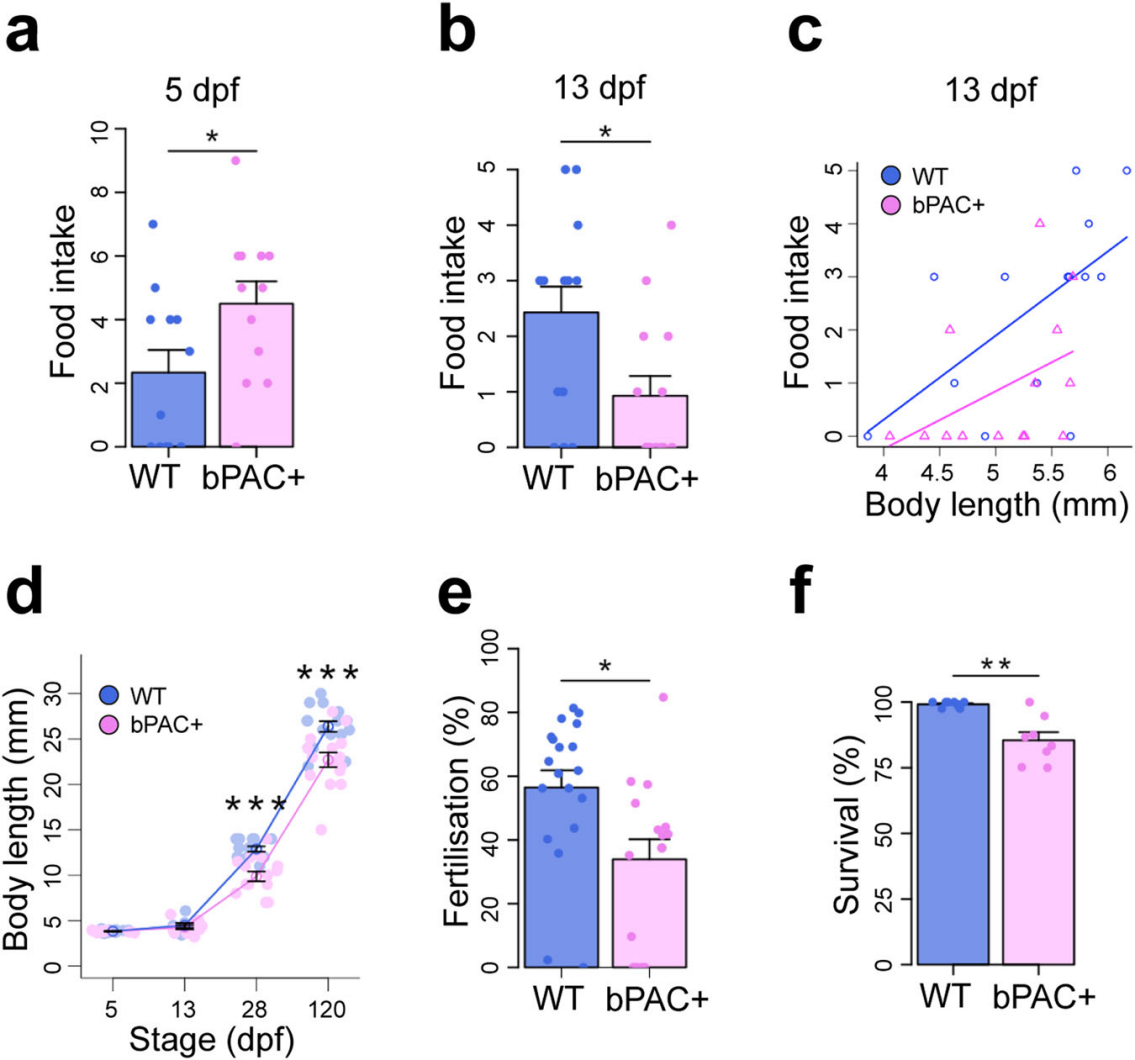
Functional consequences of elevated GC: precocious feeding followed by physical decline. **a** Analysis of feeding behaviour reveals that 5 dpf star:bPAC+ larvae eat more (*N* = 12, *t* = −2.1694, df = 21.996, *p* = 0.04113). **b** Analysis of feeding behaviour in 13 dpf wild-type and star:bPAC+ larvae reveals that food intake is significantly reduced (*N* = 14, *t* = 2.5626, df = 24.299, *p* = 0.017). **c** In 13 dpf wild-type larvae, food intake correlates positively with body size (linear regression, R-squared: 0.3619, F: 6.805 on 1 and 12 DF, *p* value: 0.02286), however in star:bPAC + , there is no significant correlation (R-squared: 0.1877, F: 2.773 on 1 and 12 DF, *p* value: 0.1218). **d** star:bPAC+ larvae are significantly smaller than wild types at 28 dpf and 120 dpf, but not at 5 dpf or 13 dpf (Two-way ANOVA with Tukey’s test, ***p < 0.001 for 28 and 120 dpf, 5 dpf: *N* = 12, 13; 13 dpf *N* = 9, 10; 28 dpf *N* = 13, 15; 120 dpf *N* = 15,15 for wild type and bPAC+ respectively). **e** Percentage of embryos that were fertilised by adult male star:bPAC+ fish is significantly lower compared to wild types (*N* = 19 wild type, *N* = 16 bPAC+ pairs, *t* = 2.7181, df = 31.072, *p* = 0.01064). **f** Percentage of star:bPAC+ fish that survived to 2 months is significantly reduced, compared with wild types (Wilcoxon test, W = 65.5, *p* = 0.004315, *N* = 9 wild type *N* = 8 bPAC+ stocks).

The increased size and proliferative capacity of the hypothalamus in 5 dpf star:bPAC+ larvae suggested that development of the hypothalamus is accelerated in these animals. Therefore, we hypothesised that behaviours associated with the hypothalamus that develop around this developmental stage might also be altered and thus we analysed feeding in 5 dpf larvae. Feeding usually emerges at around 120 hpf (5 dpf) in lab-raised zebrafish larvae, and larvae are capable of eating small live food. We exposed 5 dpf larvae to live rotifers and saw that star:bPAC+ larvae consumed significantly more rotifers during the trial (Fig. 5a, Supplementary video 1), suggesting that GC-induced precocious hypothalamic development is accompanied by early emergence of an associated behaviour, feeding.

In stark contrast to 5 dpf, at 13 dpf star:bPAC+ larvae fed less than wild types (Fig. 5b). Interestingly, in 13 dpf wild-type larvae we observed that food intake positively correlated with body size, in that larger larvae ate more, whilst in star:bPAC+ larvae no significant trend was apparent. (Fig. 5c). Hence, star:bPAC+ larvae consumed less, regardless of their size. Whilst at 5 dpf and 13 dpf we did not observe any difference in larval body size, at 28 dpf and 120 dpf star:bPAC+ fish were significantly smaller than wild types (Fig. 5d). This suggests that the reduction in food intake in star:bPAC+ animals from 13 dpf ultimately leads to impaired growth. Finally, we also observed reduced fertilisation rates and reduced long-term survival of star:bPAC+ fish (Fig. 5e, f). Together, these results point towards potential functional consequences of failed hypothalamic development after 5 dpf, including impaired behaviour and an early physical decline. Further work is required to directly link altered hypothalamic neurogenesis with the observed functional alterations in GC-exposed animals.

## Discussion

This work shows that elevated glucocorticoid alters the trajectory of hypothalamic development and function. We identify that the hypothalamus is a highly GC-sensitive region where elevated GC causes precocious development followed by failed maturation and early decline accompanied by impaired feeding, growth, and survival.

Our data supports that, at least in some cases, reduced cell proliferation following GC exposure might be related to a prior increase in proliferation. This aligns with the concept of GC-inducible stem cells^31,32^. Previous studies have demonstrated GC-induced proliferation in various contexts^33–36^, resembling mechanisms observed during brain injury, where quiescent radial glia enter the cell cycle in response to trauma^29,37^. Few studies have explored the developmental impact of GC on cell proliferation. Noorlander et al. observed that antenatal GC treatment in mice led to an initial reduction in embryonic hippocampal proliferation, followed by increased proliferation postnatally and subsequent reduction in adulthood^38^. Similarly, mouse models of ELS exhibited an initial increase in hippocampal proliferation followed by a later decline^10,12^. A meta-analysis on age-dependent effects of ELS indicated a negative correlation between age and changes in proliferation, suggesting that ELS-exposed animals exhibit increased proliferation early in development, followed by a reduction in later life^39^. It is possible that our interpretation of the results presented here also applies to other studies, in that GC drives precocious proliferation leading to failed maturation and a rapid decline.

In our model, we observed profound effects on a population of Neural stem/progenitor cells (NSPCs) within the proliferative zone of the developing hypothalamus. This population of hypothalamic radial glia might be akin to tanycyte cells observed in mammals, which also express *Rax*/*rx3* and *Blbp*^18^, and were recently shown to be sensitive to ELS exposure in adult mice^17^. We postulate that these cells might be especially sensitive to elevated GC, since they are adjacent to the 3rd ventricle, potentially detecting elevated GC levels within the CSF. The increased sensitivity of the hypothalamus to GC could also be influenced by higher levels of GR, however, it remains unclear whether the zebrafish hypothalamus expresses higher GR levels than other brain regions. The specificity of the proliferation phenotype observed in our study lacks a clear explanation. While it’s uncertain if the hypothalamus is uniquely affected in our model, it’s plausible that GC effects on different brain regions may emerge over different time scales. Previous research injecting cortisol into zebrafish at the 1-cell stage, mimicking maternal cortisol transfer, demonstrated increased neurogenesis in specific brain regions, such as the pallium and preoptic region, but not in the rostral hypothalamus of larvae at 5 dpf^40^. This supports that GC can induce neurogenesis in specific brain regions, likely depending on the context of the exposure.

Developmental GC exposure in star:bPAC+ larvae significantly affected feeding behaviour, with increased consumption at 5 dpf and decreased consumption at 13 dpf, persisting into adulthood^21^. These changes correlated with an early increase in proliferative hypothalamic radial glia at 5 dpf, diminishing by 13 dpf, accompanied by a reduction in feeding-regulating neurons expressing *agrp*, *cart*, and *hcrt* at 13 dpf. In larval zebrafish, hypothalamic *agrp* neurons are known to stimulate feeding^41,42^, and orexin, produced by *hcrt* neurons, is also known to stimulate food intake in zebrafish^43^. Meanwhile the effects of *cart* on appetite are more complex^44^. We also observed an increase in *pmch*+ neurons at 13 dpf. Whilst the role of MCH is not fully understood in zebrafish, its expression is known to increase in response to fasting in zebrafish^45^, and in goldfish it is anorexigenic^46^. As such, it is likely that the observed reduction in numbers of *agrp* and *hcrt* neurons and increase in *pmch* neurons in 13 dpf star:bPAC+ larvae contribute to the observed reduction in food consumption at the same developmental stage. At 5 dpf, increased feeding in star:bPAC+ larvae coincided with reduced *crh*+ neurons and an increase in *npy*+ neurons. Although CRH neurons can stimulate food intake^47^, evidence indicates that in the arcuate nucleus CRH may inhibit appetite-stimulating *agrp* + /*npy*+ neurons, thus stimulating food intake^48^. Thus, in our model, it is plausible that reduced *crh* expression might dis-inhibit *npy* expression, leading to the observed increase in *npy*+ neurons, subsequently stimulating food intake in 5 dpf star:bPAC + . Previous work also supports that hypothalamic *th*+ neurons stimulate food intake by regulating activity of *pomc*+ and *npy* + /agrp+ neurons^49^, suggesting that the increased *th* in specific hypothalamic DA clusters may also play a role in the early increase in food consumption we observed.

Another hypothesis regarding the basis of the altered food consumption in the star:bPAC+ larvae relates to the observed changes in hypothalamic radial glia. The population of hypothalamic radial glia altered by developmental GC exposure in our model display similarities to mammalian tanycyte cells, based on location, morphology, and marker expression^18^. Recent work demonstrated that hypothalamic tanycytes can regulate food intake^50^. Hypothalamic tanycytes are known to project to neighbouring orexigenic *npy*/*agrp* and anorexigenic *pomc* neurons of the arcuate nucleus, and tanycyte activation was shown to depolarise these neurons in vitro, and lead to hyperphagia in vivo. It is interesting to postulate that if similar functionality occurs in the hypothalamic radial glia of zebrafish, then the observed increase in these cells at 5 dpf might contribute to the increased feeding behaviour observed, meanwhile the subsequent reduction of these cells might contribute to the reduction of feeding behaviour observed in 13 dpf star:bPAC+ larvae. Further work is required to characterise the development and function of the zebrafish hypothalamic radial glia, and to determine the mechanisms underlying the altered feeding observed in star:bPAC+ larvae. Whilst the reduced food intake in 13 dpf star:bPAC+ larvae potentially contributes to the subsequent decline in growth, activation of GR signalling is known to supress growth via increased protein catabolism, and this is potentially also a contributing factor in our model^51^.

**Fig. 6:**
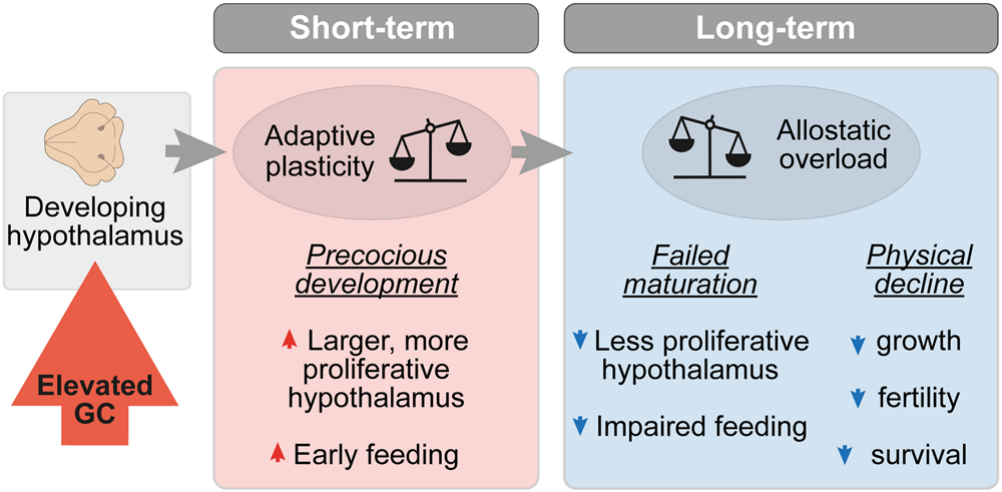
Model proposing how elevated GC alters the trajectory of hypothalamic development and function. In our model of elevated GC, we suggest that in the short-term, GC exposure drives adaptive plasticity, whereby we observed precocious hypothalamic development. In the long-term, chronic GC exposure leads to an accumulation of allostatic load, leading to allostatic overload. We observed this via failed hypothalamic maturation during the larval stage, and subsequent early physical decline of exposed fish.

We hypothesise that the precocious hypothalamic development observed here may represent an example of GC-induced adaptive plasticity (Fig. 6), as observed in other animals. For example in red squirrels, females exposed to high-density cues (mimicking increased competition) increase their levels of endogenous GC which drives them to produce offspring that grow faster than controls^52^. This so-called adaptive plasticity is acknowledged to have short-term fitness benefits, but the investment is often associated with costs in later life^53^. The costs of accelerated development can include reduced lifespan, which is known as the growth-rate lifespan trade-off^54^, as well as reductions in breeding success^55^. Interestingly, there is evidence directly linking divergence from the normal growth trajectory with lifespan, since sticklebacks that are forced to grow faster have a reduced lifespan, whilst those in which growth was slowed down lived longer than controls^54^. In our model, chronic GC exposure ultimately appears to lead to allostatic overload, a maladaptive state whereby an organism can no longer adapt to its conditions^56^. Whilst GC-induced acceleration of growth-rate leading to adverse phenotypes later is widely observed at the organism level, the underlying cellular and molecular mechanisms are poorly understood. Our work provides new insight into these processes in the developing brain. However, the potential evolutionary benefit of the precocious hypothalamic development and early feeding observed in our study requires further investigation.

In the context of the early-late life trade-off, the subsequent reduction in breeding success and/or lifespan is thought to be related to oxidative stress^57,58^. Exposure to elevated GC is known to increase oxidative stress^59^, a phenomenon in which an over-accumulation of reactive oxygen species (ROS) occurs in cells and tissues^60^. ROS is known to cause cellular damage, especially to DNA, and can lead to accelerated telomere shortening^61^. In a previous study of amphibian larvae, the relationship between stress, development, growth and aging depended on the type of stressor exposure^62^. Predator exposure initiated faster development and enhanced growth of survivors, but they showed signs of oxidative stress, had shorter telomeres and reduced long-term survival^62^. This indicates that stress exposure may initiate faster development in early life, but at a cost to long-term health and lifespan.

Telomeres shorten with every round of cell division and when a critical size is reached this imposes a functional limit on cell replication, leading to replicative senescence, which can contribute to age-related diseases^61^. One possibility in our model is that the excess proliferation of hypothalamic progenitors observed in 5 dpf star:bPAC+ larvae leads to subsequent senescence of those cells. In contrast to quiescent, apoptotic, or terminally differentiated cells, senescent cells have incurred an irreversible cell cycle arrest, yet remain viable, have alterations in metabolic activity and undergo dramatic changes in gene expression^63^. In support of our hypothesis, a previous study of liver progenitor cells reported that GC exposure induced cell proliferation leading to long-term replicative senescence/stemness exhaustion^64^, and GC is also reported to induce senescence of NSPCs in vitro^65^. The presence of senescent cells in the proliferative zone of the GC-exposed hypothalamus at 13 dpf might explain why we observed reduced expression of Pcna and *rx3*, and a reduced proliferative capacity of the remaining glial cells in the proliferative zone of star:bPAC+ larvae. Telomere shortening and senescence are associated with aging and age-related diseases; and models of accelerated aging present with phenotypes such as reduced survival and physical decline^66^, as observed in our model. Additionally, chronic exposure to GC is associated with signs of accelerated aging at a cellular level^67^. Further analysis of oxidative stress and telomere homoeostasis would provide further insight into the mechanisms underlying the phenotypes observed here.

We conclude that the phenotypes observed in our optogenetic model are the result of exposure to elevated GC produced in the interrenal, rather than other interrenal steroid hormones, which are not detectable in larval zebrafish^21,23,68^. We propose that GC-induced alteration of hypothalamic development observed in our model is mediated by GR-regulated gene transcription. In support of this, we showed that treatment with GR antagonist RU-486 reduced pH3+ mitotic cells in the star:bPAC+ hypothalamus. Whilst treatment with spironolactone had no effect on proliferation, whether spironolactone acts as an MR antagonist in fish, as it does in humans, is unclear^69^. GR expression in the brain is relatively ubiquitous^70^, however, in the mouse, MR expression is mostly restricted to neurons and some astrocytes of the hippocampus and is not expressed in NSPCs^71,72^, supporting that MR is unlikely to mediate the altered cell proliferation observed in our model. Conversely, MR is known to play a role in differentiation. MR overexpression stimulates neuronal differentiation^71,73^ and MR is known to play a role in hippocampal neuron fate and associated behaviours^74^. As such, it is possible that MR mediates the altered hypothalamic neuron phenotypes and behavioural changes observed in our model, however, this requires further investigation. We also provide evidence for regulation of hypothalamic *rx3* by GR. Indeed, GR signalling is known to regulate cell cycle progression in NSPCs by inducing a specific pattern of DNA methylation during aging^75^. In a model of replicative senescence, GC exposure lead to altered methylation of GR target gene *fkbp5*, which was exacerbated by age, and the subsequently increased *fkbp5* expression was associated with inflammation and myocardial infarction in humans, suggesting that GC is linked with age-related disease via a mechanism involving epigenetic regulation of *fkbp5*^76^. Meanwhile in cancer cells, GC exposure can induce a cell dormancy state which is in part senescence and in part quiescence, mediated by GR target gene *CDNK1C* (*cyclin-dependent kinase inhibitor 1C)*^77^, further supporting that GC exposure might regulate cell proliferation in our model via GR-mediated regulation of cell cycle.

We speculate that the effects of elevated GC on the hypothalamic developmental trajectory reported here might be an example of allostatic overload. Allostasis is the process of activating adaptive mechanisms in response to external or internal changes, however prolonged exposure to stressors can lead to accumulation of so-called allostatic load, and subsequently a maladaptive state of allostatic overload^8^, which is associated with age-related diseases and reduced lifespan. In support of this, it has been proposed that effects of stress or GC on NSPCs in young individuals may affect their renewal potential in the long-term, predisposing to adult disease^31^. We hypothesise that whilst precocious hypothalamic development induced by GC might be an example of adaptive plasticity in the short-term, the cost of this may be paid in later life through subsequent replication senescence of those cells, leading to failed hypothalamic maturation, subsequent impaired feeding behaviour and ultimately physical decline (Fig. 6). Further understanding of how stress and GC exposure can alter developmental trajectories at the molecular and cellular level is of critical importance to reduce the burden of mental and physical ill health across the life-course.

## Methods

### Zebrafish husbandry and maintenance

Zebrafish experiments were performed at the University of Exeter Aquatic Resource Centre and at the Johannes Gutenberg University of Mainz in compliance with local and national animal welfare laws, guidelines, and policies and approved by local government (Landesuntersuchugsamt Rheinland-Pfalz, Germany – 23 177-07/G20-1-033 and  UK Home Office PPL number: PEF291C4D). *Tg(2kbStAR:bPAC-2A-tdTomato)*^*uex300*^ were bred with wild-type TU zebrafish strain and were screened at 4 dpf on a fluorescent stereomicroscope. Adult zebrafish were maintained under standard zebrafish husbandry conditions, under a 12:12 light/dark cycle^78^. Transgenic fish exhibited normal sex ratios and displayed no gross developmental abnormalities. Until 5 dpf larvae were maintained at 50 larvae per Petri dish in 40 ml system water, after which they were maintained until 13 dpf in 200 ml system water. For most experiments, larvae at 5 dpf, 8 dpf, 13 dpf, 28 dpf were used and sex is not yet determined. For fertilisation rate only males were used. For body size analysis at 120 dpf, equal numbers of males and females were used.

### RNA sequencing

RNA sequencing data was obtained from our other study^21^, which is deposited in the European Nucleotide Archive (ENA, PRJEB53713). The run accession IDs for the RNA-seq data reported in this study are: TU WT 6 dpf: ERR10476787 - ERR104767 91, *star*:bPAC positive 6 dpf: ERR10476 807 - ERR10476 811; TU WT 13 dpf: ERR104767 92 - ERR104767 96; *star*:bPAC positive 13 dpf: ERR10476 812 - ERR10476 816. The detailed protocol for sample collection, RNA preparation, mRNA sequencing, and bioinformatic analysis is available in an online methods repository^79^.

### Gene ontology analysis

The functional enrichment analysis was performed using g:Profiler (version *e107_eg54_p17_bf42210)* with an adjusted *p* value of 0.05. For GO analysis of neurogenesis gene clusters (Fig. 1e), significant GO terms for each cluster were manually slimmed according to QuickGO ancestor charts. Heatmaps were generated in R using the heatmap.2 function.

### qPCR

Fish were immobilised using ice-cold water and larvae or dissected juvenile/adult brains were stored in RNA later solution. Sample collection was carried out between 08:00 and 10:00. Larvae were subsequently dissected, such that each replicate consisted of 15 larval heads (eyes and jaw removed) or 3 juvenile/adult brains. RNA was extracted using TRIzol™ Reagent (Invitrogen™, 15596026), as previously described^80^, and cDNA was synthesised using High-Capacity RNA-to-cDNA™ Kit (Applied Biosystems, 4387406). Approximately 100 ng cDNA was used in a 10 μl qPCR reaction with PowerUp™ SYBR™ Green Master Mix (A25778, Applied Biosystems). Primer sequences can be found in Supplementary Data 2. The reactions were run in Hard-Shell 96-Well PCR Plates (HSP9601, BioRad) on a CFX96 Real Time PCR machine (Bio-Rad) using a standard protocol. Relative expression was calculated using the 2^–∆∆Ct^ method, with *18* *s* or *sep15* as a reference gene.

### Fluorescent in situ hybridisation (FISH) and immunohistochemistry (IHC)

The FISH and IHC methods were based on a protocol by Jakob von Trotha (2016) for whole zebrafish embryos and larvae. Larvae or juvenile fish were immobilised in ice-cold water and then whole larvae or dissected juvenile fish brains were fixed in 4% PFA overnight at 4 °C, dehydrated in methanol and subsequently stored at −20 °C. After rehydration, larvae or brains were permeabilised using proteinase K (10 μg/ml) treatment at 37 °C and then re-fixed in PFA. DIG-conjugated mRNA probes were hybridised overnight at 65 °C. In situ hybridisation probes for *rx3*, *agrp*, *cart4, npy, pmch, th, crhb, avp* were synthesised using primers listed in Supplementary Data 2. *pomc* probe was synthesised from a plasmid, as previously described^81^. After removal of probe and washing, samples were blocked for 2 h in blocking solution (1% blocking reagent; (Roche, 11096176001) in malic acid buffer (0.15 M maleic acid, 0.15 M NaCl, PH 7.5)) and then incubated overnight at 4 °C in anti-DIG POD antibody (Roche, 11207733910) diluted 1:300 in blocking solution. After more washing, samples were incubated for 40 min in the dark with 1:200 FITC-tyramide (synthesised from product 46410, Thermo Scientific), 0.003% H2O2, 2% dextran sulphate in PBST. For IHC antigen retrieval was performed according to published methods^82^. Samples were blocked in 10% Normal goat serum in PBST for 2 h before incubation in primary antibody overnight at 4 °C. For IHC of BrDU-treated samples, samples were treated with 1 N HCl for 30 min prior to blocking. Primary antibodies used were: pH3 (Merck/Millipore, 06-570), Pcna (Sigma, MABE288), BLBP (Sigma, ABN14), Otpa^83^, BrDU (abcam, ab6326). After washing all day, samples were incubated overnight in 1:1000 secondary antibody with 1:200 hoechst (H3570, Invitrogen). Secondary antibodies used were Goat anti-Rabbit IgG (H + L) Cross-Adsorbed Secondary Antibody, Alexa Fluor™ 488 (A-11008, Invitrogen), Goat anti-Rabbit IgG (H + L) Cross-Adsorbed Secondary Antibody, Alexa Fluor 633 (A-21070, Invitrogen), Goat anti-Mouse IgG (H + L) Cross-Adsorbed Secondary Antibody, Alexa Fluor 568 (A-11004, Invitrogen), Goat anti-Rat IgG (H + L) Cross-Adsorbed Secondary Antibody, Alexa Fluor 488 (A-11006, Invitrogen). After staining all samples were washed further then cleared overnight in glycerol. Samples were imaged on a Zeiss LSM 880 confocal microscope using a 25x or 10x objective.

### Image processing

Image processing was performed in Fiji (ImageJ2, version 2.9.0). For whole brain counting of cells, z-stacks were taken across the brain from dorsal to ventral and regions were defined using the hoechst staining with the Z-Brain viewer^84^. Images were thresholded and pH3 cells in each region were manually counted in a blinded manner. For counting of cells within the hypothalamus, cell counts were performed manually from z-stacks, in a blinded manner. Similarly, hypothalamic volume was calculated based on hoechst staining in z-stacks, using Z-Brain viewer. Corrected Total Cell Fluorescence (CTCF) was calculated as Integrated Density of cells – (Area of cells X Mean fluorescence of background reading). For CTCF calculations, labelled cells were identified first by thresholding each image and creating ROIs of the labelled cells using the Analyze Particles function. The total Integrated density and total area of all labelled cells was then used to calculate CTCF for each sample. For co-expression analysis, ROIs of labelled cells were generated for each marker as described above. Percentage overlap was calculated using the Analyze Particles function with ROIs of marker 1 cells against a mask of marker 2 cells. Total area of cells was calculated from the thresholded mask/ROIs of labelled cells. pH3 cells were manually scored as *rx3*- or *rx3*+ using the ROIs/masks as described above, in a blinded manner. All measures are normalised relative to size of the hypothalamus. For the BrDU experiment, BrDU+ and Pcna+ cells are reported as the area of labelled cell coverage in μM. This was calculated from the thresholded fluorescent signal for each marker within the hypothalamus.

### Feeding behaviour

Prior to behavioural analysis, larvae were transferred to 24 well cell culture plates (83.3922, Sarstedt) with 1 larva per well in 1.5 ml aquarium water. Larvae were then left to acclimate to the behavioural testing room (maintained at 28 °C) for 2 h. 5 dpf larvae were fed 1 ml of diluted live rotifers (approximately 20), meanwhile 13 dpf larvae were fed 5 live artemia. Larvae were recorded for 10 min using Basler Video Recording Software. The camera used was a Basler (Germany) acA1300-200um USB 3.0 camera with ON Semiconductor PYTHON 1300 CMOS sensor (203 frames per second at 1.3 MP resolution) with a Computar Zoom Lens 18-108/2.5 (Japan). Number of successful and unsuccessful prey captures and latency to first hunting attempt were analysed manually from video recordings in a blinded manner.

### BrDU experiment

Larvae were transferred to a 12 well dish with 10 larvae per well. Larvae were incubated overnight from 4 dpf in 3 ml 10 mM 5-Bromo-2′-deoxyuridine (B5002, Sigma) with 1% DMSO for 17 h. On the morning of 5 dpf, BrDU was removed by washing 3-times with aquarium water. Larvae were subsequently fixed in PFA or raised until a later stage for fixation.

### Drug treatments

Larvae were incubated from 6 hpf until 120 hpf with 50 μM dexamethasone (Sigma-Aldrich D2915) or from 48 hpf until 120 hpf with 2 μM RU-486 (Sigma-Aldrich, M8046) or with 10 μM Spironolactone (Sigma-Aldrich S3378). DMSO solution was used as a control with 0.008% DMSO for Spironolactone control and 0.004% DMSO for Mifepristone control. Solutions were changed daily. Dexamethasone concentration was determined based on a previous study that found that 50 μM dexamethasone could reliably induce negative feedback to the HPA axis^85^. RU-486 concentration was selected based on a previous study of GR mutant zebrafish^86^. The spironolactone concentration used was selected based on a previous study^87^.

### ChIP-qPCR

We analysed the sequence for the *rx3* gene (ENSDARG00000052893, chromosome:GRCz11:21:10755554:10759823:1). Primers for *rx3* GRE1 and GRE2 are described in supplementary data 2 and were designed using Primer3. GR bounded chromatin was prepared following the protocol from Idilli et al.^88^, with minor modifications. For each sample, 100 dissected 5 dpf larval heads (without eyes) were incubated for 10 min at room temperature in 1% formaldehyde in PBS with protease inhibitors (A32955, Thermo Scientific) for cross-linking. We used a glucocorticoid receptor polyclonal antibody (24050-1-AP, Proteintech) and Rabbit IgG Isotype Control (10500 C, Invitrogen) as primary antibodies. For each sample, 3 technical replicates were run on the PCR machine.

### TUNEL assay

Apoptotic cells were labelled using the In Situ Cell Death Detection Kit, Fluorescein (11684795910, Roche). Larvae were first fixed, dehydrated, rehydrated and permeabilised as per the FISH protocol. Larvae were incubated in TUNEL reaction mix at 37 °C for 2 h and subsequently, washed and cleared as per the FISH protocol.

### Fertilisation analysis

Fertilisation rate of adult star:bPAC+ males was determined during standard pair matings with wild type females and compared with wild type crosses. The fish used were 11 months of age. Embryos were collected from 19 wild type and 16 bPAC+ pairs across 3 independent mating trials and successful fertilisation was calculated at 6−8 hpf by identifying the number of unfertilised embryos in each Petri dish. The fertilisation rate for each pair was determined by averaging across all Petri dishes of embryos that came from each pair. Survival of fertilised embryos was confirmed at 24 hpf.

### Survival analysis

Survival of star:bPAC+ and wild type fish was determined by calculating the number of fish that survived in a stock from when larvae were placed in the facility nursery at 5 dpf until 2 months of age when fish were prepared for transfer to the main aquarium facility. Survival was calculated from 9 wild type and 8 bPAC+ stocks that were placed into the nursery, where each stock consisted of between 40 and 300 fish that were housed across multiple tanks at the same stocking density. These data were collected from different stocks that were raised over a duration of more than 2 years.

### Cortisol

A step-wise method is reported in an online repository^79^. On the day prior to cortisol analysis, groups of 12 larvae were pipetted into each well of a 6-well plate with 6 ml system water per well. For filtered light treatments the plate was stored inside a custom-built box covered by 550 nm long-pass filters (Thor- labs) and sampling was performed under filtered light. For acute light exposure of filter-raised larvae, the lid was removed from the filter box whilst the box is inside the lit incubator, and larvae were exposed to white light for 10 min. Whilst still inside the incubator, larvae were immobilised using ice water, transferred to an Eppendorf, and the water was removed. Samples were snap frozen on liquid nitrogen. The cortisol assay (Cisbio HTRF Cortisol Kit, 62CRTPEG) was performed following the manufacturer’s protocol. Signal was detected using a CLARIO star plate reader (BMG Labtech).

### Statistics and reproducibility

All analyses and graphs were generated in R (RStudio 2022.07.2). Prior to testing for statistically significant differences between groups, data were tested for normality and variance. Where data did not fit the assumptions for t-testing or ANOVA, non-parametric alternatives were used. Where appropriate, Bonferroni correction for multiple testing was used. In all bar graphs mean ± standard error is presented for each group. Sample sizes consisted of between 4 and 23 biological replicates per group for microscopy experiments. For the RNA sequencing experiment, there were 5 pools of brain samples per group. For cortisol experiments there were between 5 and 9 pools of larvae per group. For ChIP-qPCR there was 1 pool of biological replicates per group, run as 3 technical replicates. For functional studies there were between 8 and 19 replicates per group. Exact sample sizes and number of replicates are indicated in each figure legend.

### Reporting summary

Further information on research design is available in the Nature Portfolio Reporting Summary linked to this article.

### Supplementary information

Supplementary Information

Description of Additional Supplementary Files

Supplementary data 1

Supplementary Data 2

Supplementary Video

Reporting summary

## Data Availability

All sequenced reads for RNA-seq were deposited in European Nucleotide Archive as part of our other study^21^ (ENA, PRJEB53713). The run accession IDs for the RNA-seq data reported in this study are: TU WT 6 dpf: ERR10476787 - ERR104767 91, *star*:bPAC positive 6 dpf: ERR10476 807 - ERR10476 811; TU WT 13 dpf: ERR104767 92 - ERR104767 96; *star*:bPAC positive 13 dpf: ERR10476 812 - ERR10476 816. Source data for figures are available in Supplementary data file 1. All other data are available from the corresponding author on reasonable request.

## Code availability

The code used for bioinformatic analysis of the RNA sequencing data is available in an online methods repository^79^.
