# Supplementary material for: Elevated glucocorticoid alters the developmental dynamics of hypothalamic neurogenesis in zebrafish
Source: Commun Biol. 2024 Apr 5;7:416. doi: 10.1038/s42003-024-06060-5 (PMC10997759; doi:10.1038/s42003-024-06060-5)
Supplement: Supplementary file 1 — Supplementary Information [file 42003_2024_6060_MOESM1_ESM.pdf]

Supplementary Figure 1

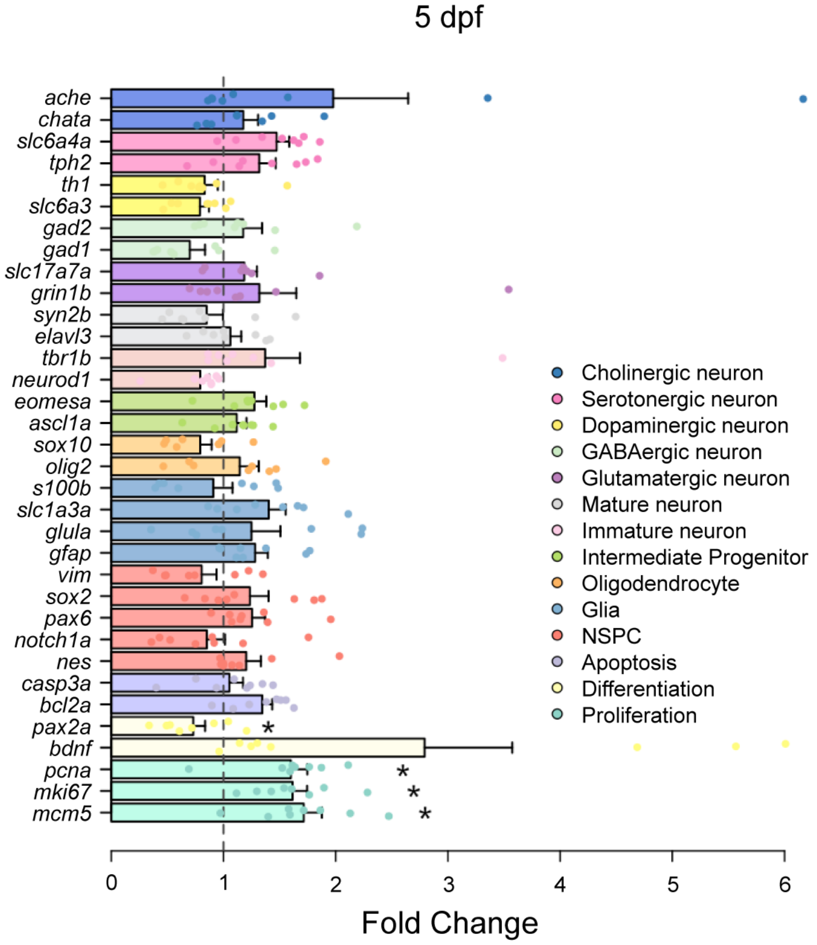

Cell proliferation is increased in brains of 5 dpf star:bPAC+ larvae.

qPCR analysis of 5 dpf whole brain samples indicates a significant increase in mRNA expression of three genes associated with cell proliferation (*pcna*, *mki67*, *mcm5*) and one gene associated with cell differentiation (*pax2a*) in star:bPAC+ whole brain samples, compared with wild types. Fold change expression (relative to reference gene *sep15*) in star:bPAC+ relative to wild types is shown (N=8 pools of 12 heads/sample, * $p<0.05$).

Supplementary Figure 2

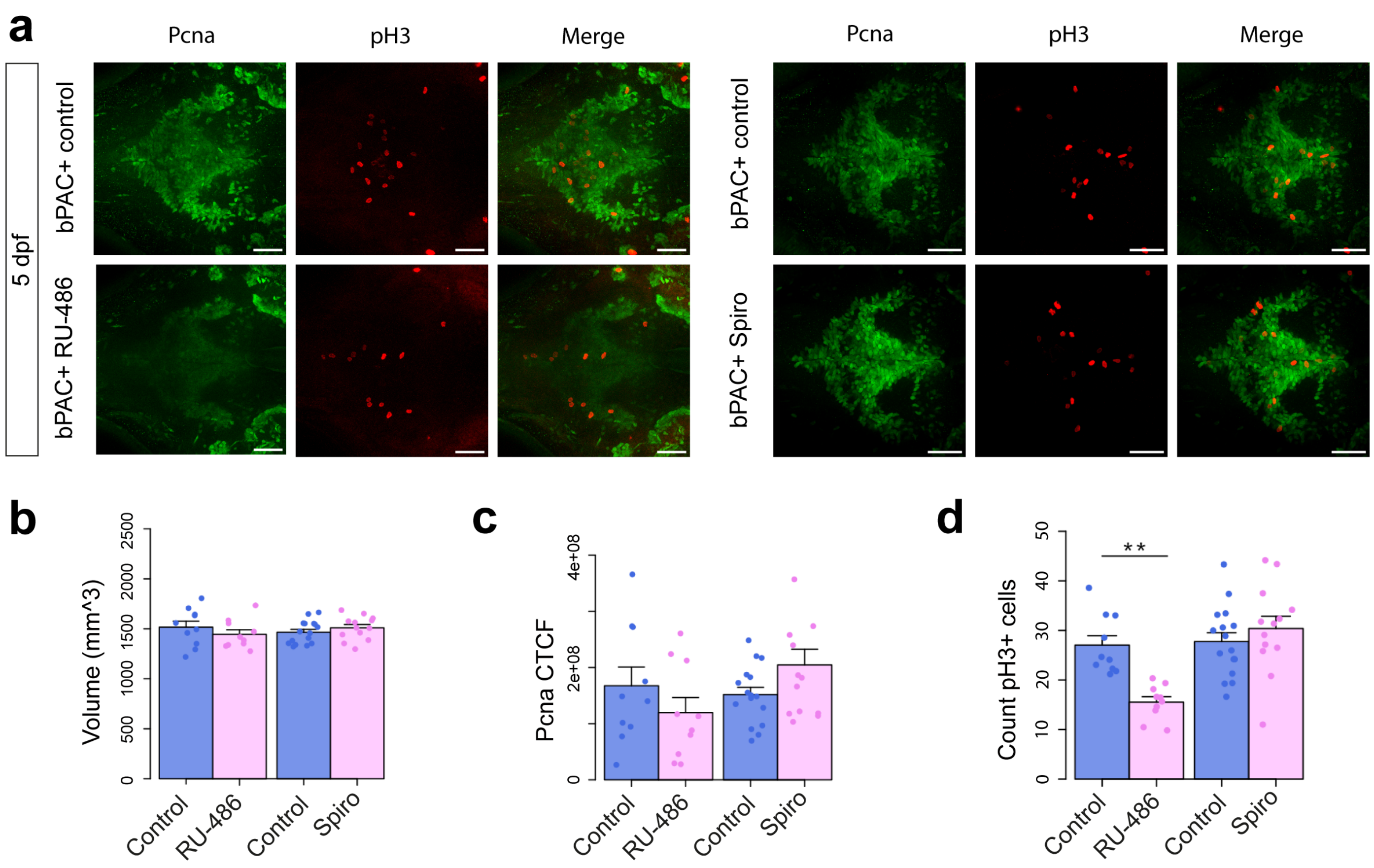

Hypothalamic proliferation is regulated by GR during development.

a. Maximum intensity projection of a confocal stack through the hypothalamus of 5 dpf star:bPAC+ control fish and following treatment with RU-486 or Spironolactone, with pH3 (red) and Pcna IHC (green). Scale bar is 40 μm. **b.** Hypothalamus volume is not affected by RU-486 or Spironolactone treatment in 5 dpf star:bPAC+ larvae (ANOVA, F=0.692, df=3,45, p-value= 0.562). **c.** Normalised Pcna CTCF in the hypothalamus is not significantly affected by RU-486 or Spironolactone treatment in star:bPAC+ larvae (ANOVA, F=1.988, df=3, 45, p-value= 0.129). **d.** Normalised count of pH3+ cells is significantly reduced by RU-486 but not by Spironolactone treatment in the hypothalamus of 5 dpf star:bPAC+ fish (ANOVA followed by Tukey post-hoc test, F=9.83, df= 3,45, p-value= 0.000042). Graphs show the mean with standard error. N=10 for RU-486 and control; N=13 and N=16 for Spironolactone and control respectively. **, p<0.01.

Supplementary Figure 3

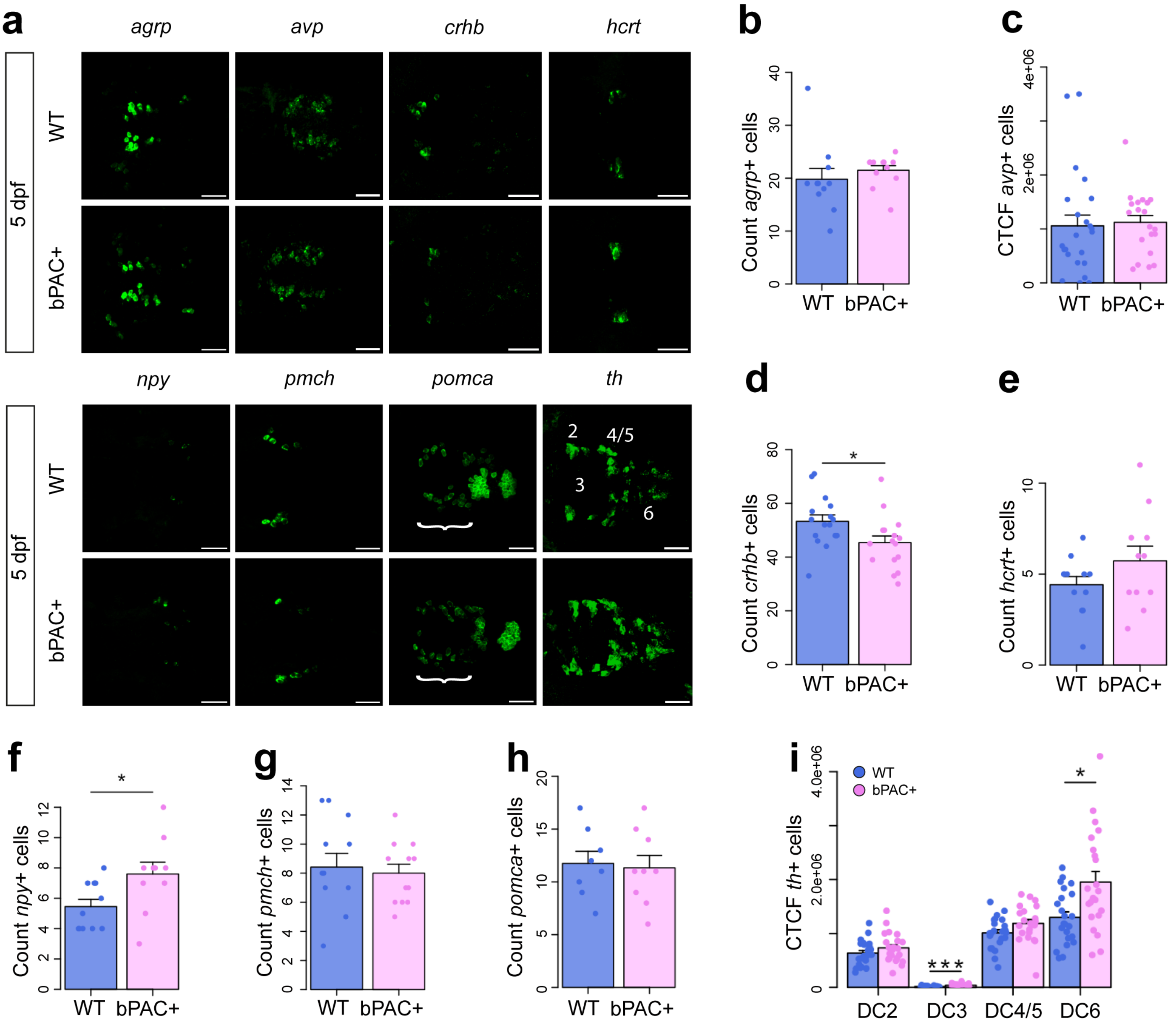

Hypothalamic neuronal subtypes in 5 dpf star:bPAC+ larvae.

a. Expression of genes associated with neuronal subtypes in the hypothalamus of 5 dpf star:bPAC+ and wild-type larvae as shown in maximum intensity projections of confocal stacks. Scale bar is 40 µm. **b.** The number of *agrp*+ cells is not different in the arcuate nucleus of 5 dpf star:bPAC+ larvae compared with wild types (N=11 WT, N=12 bPAC+, t = -0.75733, df = 13.434, p-value = 0.4619). **c.** The number of *avp*+ cells is not significantly different (N= 23 wild types, N=21 bPAC+, t = -0.280, df = 36.51, p-value = 0.78) in the ventral and rostral hypothalamus of 5 dpf star:bPAC+ larvae compared with wild types. **d.** The number of *crhb*+ cells is significantly lower in the hypothalamus of 5 dpf star:bPAC+ larvae compared with wild types. (N= 16 per group, t = 2.3127, df = 29.953, p-value = 0.02779). **e.** The number of *hcrt*+ cells is not significantly different (N=12 WT, N=11 bPAC+, t = -1.4132, df = 15.794, p-value = 0.177) in the dorso-rostral hypothalamus of 5 dpf star:bPAC+ larvae compared with wild types. **f.** The number of *npy*+ cells is significantly higher (N=11 WT, N=10 bPAC+, t = -2.356, df = 15.064, p-value = 0.03243) in the intermediate hypothalamus of 5 dpf star:bPAC+ larvae compared with wild types. **g.** The number of *pmch*+ cells is not significantly different (N= 12 per group, t = 0.37053, df = 18.954, p-value = 0.7151) in the intermediate hypothalamus of 5 dpf star:bPAC+ larvae compared with wild types. **h.** The number of *pomc*+ cells is not significantly different (N=8 WT, N=9 bPAC+, t = 0.25482, df = 14.965, p-value = 0.8023) in the arcuate nucleus of 5 dpf star:bPAC+ larvae compared with wild types. Bracket indicates hypothalamic *pomc*+ cells in the arcuate nucleus of the hypothalamus that were included in the analysis, the neighbouring pituitary *pomc*+ cells were not included. **i.** The number of *th*+ cells is significantly higher for DA clusters 3 and 6 in the hypothalamus/PT of 5 dpf star:bPAC+ larvae compared with wild types (repeated measures ANOVA followed by pairwise comparisons using Mann Whitney test with Sidak correction for multiple comparisons; genotype: region

interaction, F=6.11, d.f.=3, 109, P=0.00069, N=23 wild types, N=22 bPAC+ across 2 independent experiments). *, p<0.05; ***, p<0.001 for star:bPAC+ compared to wild type.

Supplementary Figure 4

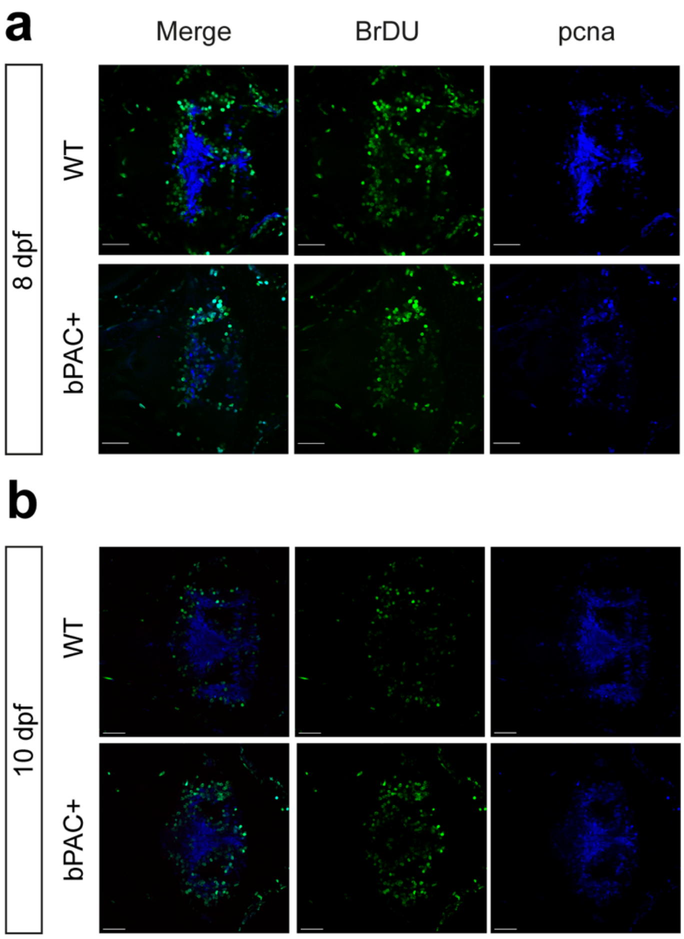

Altered differentiation in the developing star:bPAC+ hypothalamus. Confocal images of BrDU lineage tracing analysis at 8 dpf (a) and 10 dpf (b). In each panel wild types are shown in the upper row and star:bPAC+ larvae in the bottom row. The left-most column shows the overlap between BrDU-labelled cells with endogenous pcna, whilst single channel images for BrDU and pcna are adjacent. All images are a single plane from a confocal z-stack. In all cases the scale bar indicates 40 μm.

Supplementary Figure 5

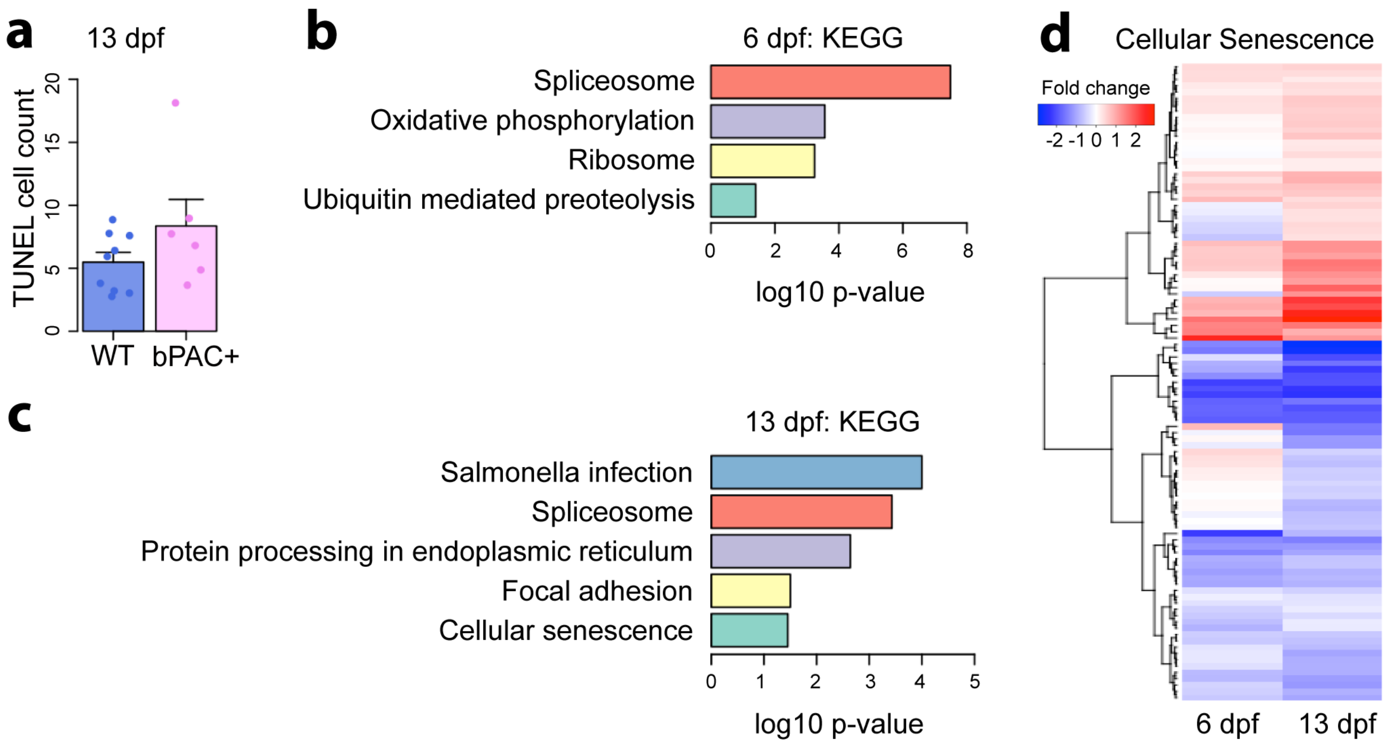

Cell death and cellular senescence in star:bPAC+ larvae.

a. The total number of TUNEL+ cells in the hypothalamus is not different in 13 dpf star:bPAC+ larvae, compared with wild types (Wilcoxon test, N=9 wild type, N=6 bPAC+, W = 16, p-value = 0.2238). **b-c**. KEGG pathway analysis of DEGs in the RNA-seq data from 6 dpf (b) and 13 dpf (c) star:bPAC+ versus wild-type larvae. Significant pathways with an adjusted p-value of <0.05 are shown. Cellular senescence genes are enriched in 13 dpf DEGs, but not 6 dpf. **d.** Heatmap of log2 fold changes of 102 DEGs associated with KEGG pathway 'cellular senescence' at 13 dpf, and their corresponding expression change at 6 dpf in star:bPAC+ brains compared with wild types.

Supplemental Figure 6

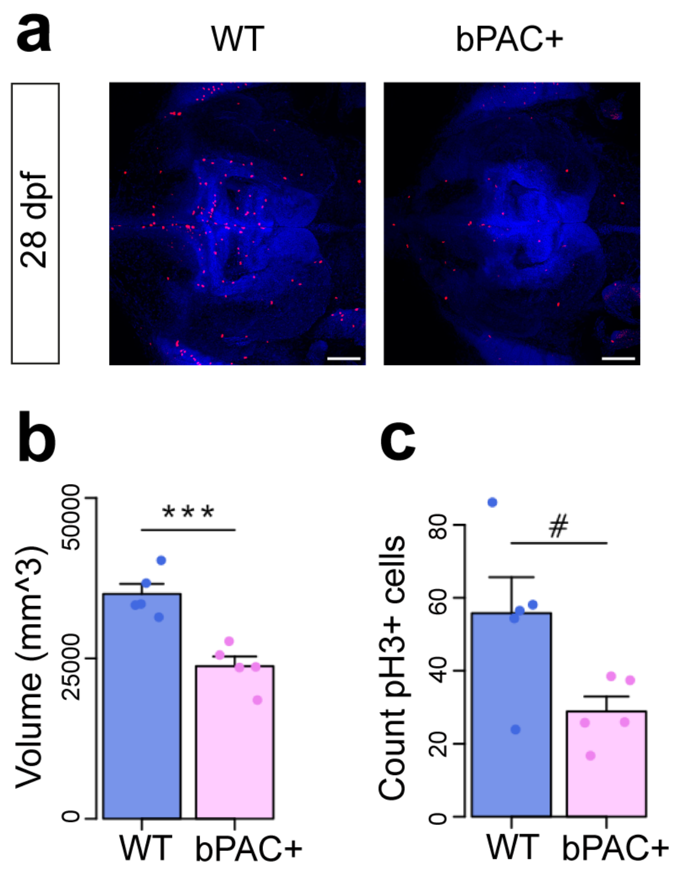

Hypothalamic proliferation is reduced in juvenile star:bPAC+ fish.

a. Maximum intensity projection of a confocal stack through the hypothalamus of 28 dpf wild-type and star:bPAC+ fish with PH3 IHC (red) and hoechst staining (blue). Scale bar is 100 µm. **b.** Hypothalamus volume is reduced in 28 dpf star:bPAC+ fish (t-test, t = 5.1649, df = 7.993, p-value = 0.0008608). **c.** Normalised count of pH3+ cells showed a trend towards a reduction in the hypothalamus of 28 dpf star:bPAC+ fish (t-test, t = 2.5237, df = 5.3203, p-value = 0.05006). Graphs show the mean with standard error. N=5. #, p<0.1, ***, p<0.001.

Supplemental Figure 7

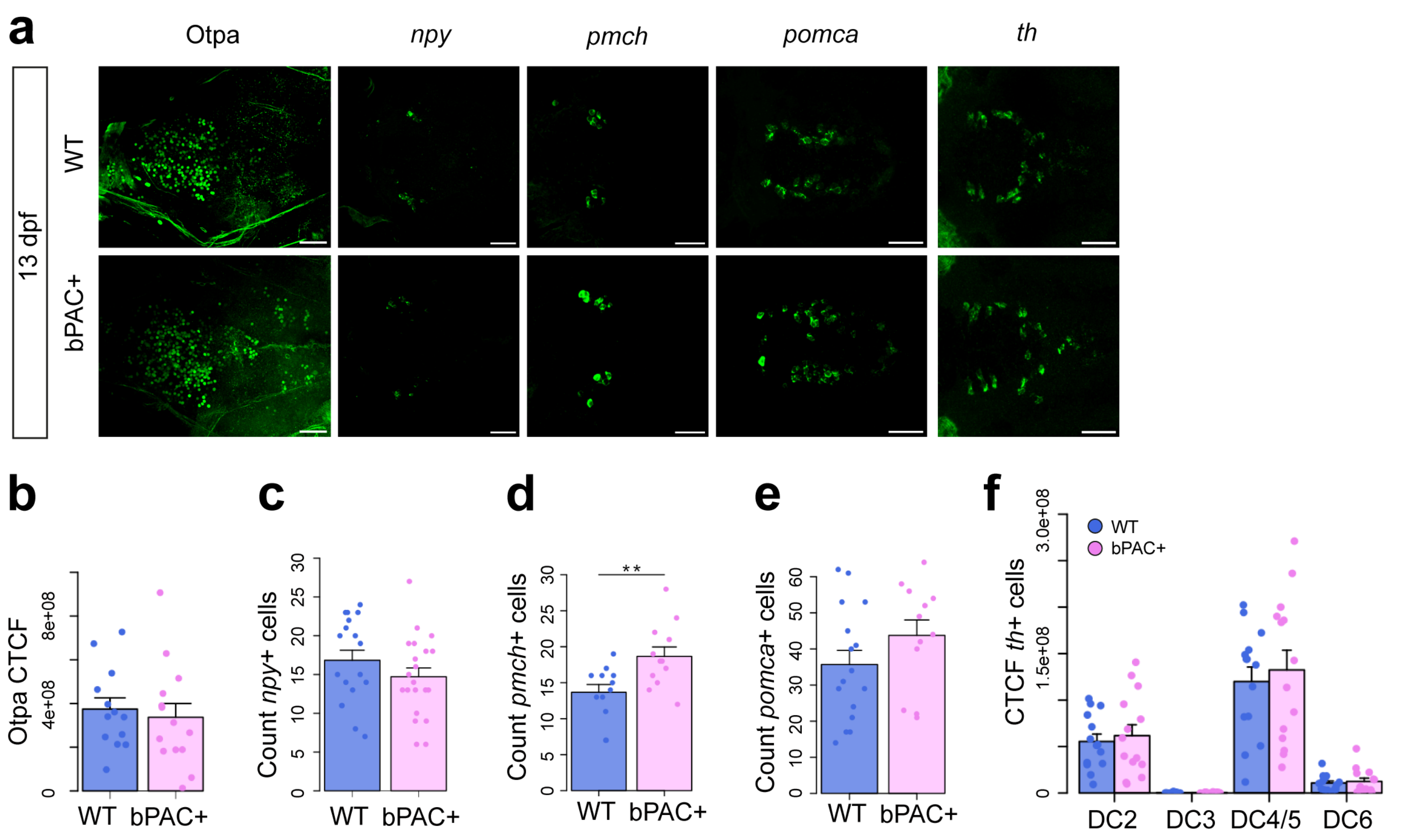

Expression of neuronal precursors and neuromodulators in the hypothalamus of 13 dpf star:bPAC+ larvae.

a. Expression of neuronal precursors and neuromodulators in the hypothalamus of 13 dpf star:bPAC+ and wild-type larvae as shown in maximum intensity projections of confocal stacks. Scale bar is 40 μm. **b.** Expression of Otpa is not different in the hypothalamus of 13 dpf star:bPAC+ larvae compared with wild types (t = 0.45864, df = 24.359, p-value = 0.6506, N=13 wild types, N=14 bPAC+) **c**. The number of *npy*+ cells is not different (N=18 wild type, N=21 bPAC+, t = 1.231, df = 35.34, p-value = 0.2264). **d.** The number of *pmch*+ cells is significantly increased in the hypothalamus of 13 dpf star:bPAC+ larvae compared with wild types (N=12, t = -2.957, df = 21.31, p-value = 0.0074). **e.** The number of *pomca*+ cells is not significantly different (N=16 wild type, N=12 bPAC+, t = -1.3905, df = 24.592, p-value = 0.1768) in the hypothalamus of 13 dpf star:bPAC+ larvae compared with wild types. **f.** The number of *th*+ cells is not significantly different in the hypothalamus/PT of 13 dpf star:bPAC+ larvae compared with wild types (repeated measures ANOVA; genotype: F=0.263, df=1,26, p=0.613; genotype: region interaction: *F*=0.179, d.f.=3, 78, *P*=0.91, N=14 per group).
