# Supplementary material for: Elevated glucocorticoid alters the developmental dynamics of hypothalamic neurogenesis in zebrafish
Source: Commun Biol. 2024 Apr 5;7:416. doi: 10.1038/s42003-024-06060-5 (PMC10997759; doi:10.1038/s42003-024-06060-5)
Supplement: Supplementary file 2 — Description of Additional Supplementary Files [file 42003_2024_6060_MOESM2_ESM.pdf]

Description of Additional Supplementary Files

File name: Supplementary Data 1

Description: Source data for all main figures.

File name: Supplementary Data 2

Description: Primer sequences for qPCR, in situ hybridisation probes and ChIP-qPCR.

File name: Supplementary Video 1

Description: Feeding behaviour in 5 dpf zebrafish larvae. Video showing larvae exposed to live food (rotifers). Two feeding events are observed for the star:bPAC+ larva (lower panel), during the clip, meanwhile the wild type larva does not feed during the clip.
