# Supplementary material for: Elevated glucocorticoid alters the developmental dynamics of hypothalamic neurogenesis in zebrafish
Source: Commun Biol. 2024 Apr 5;7:416. doi: 10.1038/s42003-024-06060-5 (PMC10997759; doi:10.1038/s42003-024-06060-5)
Supplement: Supplementary file 6 — Reporting summary [file 42003_2024_6060_MOESM6_ESM.pdf]

nature portfolio

Corresponding author(s): Soojin Ryu

Last updated by author(s): 14/02/2024

Reporting Summary

Nature Portfolio wishes to improve the reproducibility of the work that we publish. This form provides structure for consistency and transparency in reporting. For further information on Nature Portfolio policies, see our Editorial Policies and the Editorial Policy Checklist.

Statistics

For all statistical analyses, confirm that the following items are present in the figure legend, table legend, main text, or Methods section.

n/a	Confirmed	
☐	☒	The exact sample size (n) for each experimental group/condition, given as a discrete number and unit of measurement
☐	☒	A statement on whether measurements were taken from distinct samples or whether the same sample was measured repeatedly
☐	☒	The statistical test(s) used AND whether they are one- or two-sided<br>*Only common tests should be described solely by name; describe more complex techniques in the Methods section.*
☒	☐	A description of all covariates tested
☐	☒	A description of any assumptions or corrections, such as tests of normality and adjustment for multiple comparisons
☐	☒	A full description of the statistical parameters including central tendency (e.g. means) or other basic estimates (e.g. regression coefficient) AND variation (e.g. standard deviation) or associated estimates of uncertainty (e.g. confidence intervals)
☐	☒	For null hypothesis testing, the test statistic (e.g. F, t, r) with confidence intervals, effect sizes, degrees of freedom and P value noted<br>*Give P values as exact values whenever suitable.*
☒	☐	For Bayesian analysis, information on the choice of priors and Markov chain Monte Carlo settings
☒	☐	For hierarchical and complex designs, identification of the appropriate level for tests and full reporting of outcomes
☒	☐	Estimates of effect sizes (e.g. Cohen's d, Pearson's r), indicating how they were calculated

Our web collection on statistics for biologists contains articles on many of the points above.

Software and code

Policy information about availability of computer code

Data collection	The provided RNA sequencing data was collected using an Illumina NovaSeq6000. qPCR data were collected on a CFX96 Real Time PCR machine with CFX Maestro version 4.1.2433.1219. Image data was collected on a Zeiss LSM 880 confocal microscope with Zen Black version 2.3. Behavioural data was collected using a a Basler acA1300-200um USB 3.0 camera with Pylon Viewer version 7.2. Cortisol data was collected using a CLARIO star plate reader (BMG Labtech) using Reader Control software.
Data analysis	Image processing was performed in Fiji (ImageJ2, version 2.9.0). Statistical analysis and data visualisation was performed in R (RStudio 2022.07.2). Detailed description of RNA sequencing data processing and analysis is described in our online method: https://www.protocols.io/view/comprehensive-analysis-methods-for-developmental-g-kxygx9ooog8j/v3. Cortisol data was analysyed using Excel.

For manuscripts utilizing custom algorithms or software that are central to the research but not yet described in published literature, software must be made available to editors and reviewers. We strongly encourage code deposition in a community repository (e.g. GitHub). See the Nature Portfolio guidelines for submitting code & software for further information.

Data

Policy information about availability of data

All manuscripts must include a data availability statement. This statement should provide the following information, where applicable:

- Accession codes, unique identifiers, or web links for publicly available datasets
- A description of any restrictions on data availability
- For clinical datasets or third party data, please ensure that the statement adheres to our policy

RNA sequencing data was obtained from our other study (Choi et al., 2023, Biorxi 2023.02.13.528363), which is deposited in the European Nucleotide Archive (ENA, PRJEB53713). The run accession IDs for the RNA-seq data reported in this study are: TU WT 6 dpf: ERR10476787 - ERR10476791, star:bPAC positive 6 dpf: ERR10476807 - ERR10476811; TU WT 13 dpf: ERR10476792 - ERR10476796; star:bPAC positive 13 dpf: ERR10476812 - ERR10476816. All other data are available upon request.

Human research participants

Policy information about studies involving human research participants and Sex and Gender in Research.

Reporting on sex and gender	N/A
Population characteristics	N/A
Recruitment	N/A
Ethics oversight	N/A

Note that full information on the approval of the study protocol must also be provided in the manuscript.

Field-specific reporting

Please select the one below that is the best fit for your research. If you are not sure, read the appropriate sections before making your selection.

☒ Life sciences ☐ Behavioural & social sciences ☐ Ecological, evolutionary & environmental sciences

For a reference copy of the document with all sections, see nature.com/documents/nr-reporting-summary-flat.pdf

Life sciences study design

All studies must disclose on these points even when the disclosure is negative.

Sample size	All N numbers are reported in the figure legends. G*Power calculations were used to determine approximate samples sizes required for experiments where preliminary data was available to determine effect sizes. In other cases sample size was determined based on previous studies using similar methods.
Data exclusions	No data were excluded from the analysis unless a sample appeared damaged.
Replication	The number of biological replicates is indicated in the figure legend for all experiments. For cortisol analysis we performed 2 technical replicates for each biological replicate. For qPCR analysis we performed 3 technical replicates for each biological replicate.
Randomization	The order of sample collection was randomised for each experiment. Animals were randomly assigned to treatment groups.
Blinding	Data collection was not blinded. Analysis of behavioural data and image data was conducted in a blinded manner. Analysis of other data was not blinded.

Reporting for specific materials, systems and methods

We require information from authors about some types of materials, experimental systems and methods used in many studies. Here, indicate whether each material, system or method listed is relevant to your study. If you are not sure if a list item applies to your research, read the appropriate section before selecting a response.

Materials & experimental systems

n/a	Involved in the study	
☐	☒	Antibodies
☒	☐	Eukaryotic cell lines
☒	☐	Palaeontology and archaeology
☐	☒	Animals and other organisms
☒	☐	Clinical data
☒	☐	Dual use research of concern

Methods

n/a	Involved in the study	
☒	☐	ChIP-seq
☒	☐	Flow cytometry
☒	☐	MRI-based neuroimaging

Antibodies

Antibodies used

Primary antibodies used were: pH3 (Merck/Millipore, 06-570), Pcna (Sigma, MABE288), BLBP (Sigma, ABN14), Otpa (Wolf & Ryu 2013, Development), BrDU (abcam, ab6326). Secondary antibodies used were Goat anti-Rabbit IgG (H+L) Cross-Adsorbed Secondary Antibody, Alexa Fluor™ 488 (A-11008, Invitrogen), Goat anti-Rabbit IgG (H+L) Cross-Adsorbed Secondary Antibody, Alexa Fluor 633 (A-21070, Invitrogen), Goat anti-Mouse IgG (H+L) Cross-Adsorbed Secondary Antibody, Alexa Fluor 568 (A-11004, Invitrogen), Goat anti-Rat IgG (H+L) Cross-Adsorbed Secondary Antibody, Alexa Fluor 488 (A-11006, Invitrogen).

Validation

Validation for the following commercial antibodies has been performed according to the manufacturer website:
pH3: https://www.merckmillipore.com/GB/en/product/Anti-phospho-Histone-H3-Ser10-Antibody-Mitosis-Marker,MM_NF-06-570
PCNA: https://www.sigmaaldrich.com/GB/en/product/mm/mabe288
BLBP: https://www.sigmaaldrich.com/GB/en/product/mm/abn14
BrDU: https://www.abcam.com/products/primary-antibodies/brdu-antibody-bu175-icr1-proliferation-marker-ab6326.html
Anti Rabbit 488: https://www.thermofisher.com/antibody/product/Goat-anti-Rabbit-IgG-H-L-Cross-Adsorbed-Secondary-Antibody-Polyclonal/A-11008
Anti rabbit 633: https://www.thermofisher.com/antibody/product/Goat-anti-Rabbit-IgG-H-L-Cross-Adsorbed-Secondary-Antibody-Polyclonal/A-21070
Anti mouse 568: https://www.thermofisher.com/antibody/product/Goat-anti-Mouse-IgG-H-L-Cross-Adsorbed-Secondary-Antibody-Polyclonal/A-11004
Anti rat 488: https://www.thermofisher.com/antibody/product/Goat-anti-Rat-IgG-H-L-Cross-Adsorbed-Secondary-Antibody-Polyclonal/A-11006

The Otpa antibody was validated in the following study: Wolf & Ryu, 2013, Development, https://doi.org/10.1242/dev.085357

Animals and other research organisms

Policy information about studies involving animals; ARRIVE guidelines recommended for reporting animal research, and Sex and Gender in Research

Laboratory animals

Wild-type TU and Tg(star:bPAC-2A-tdTomato)uex300 zebrafish were used at 5 dpf, 8 dpf, 10 dpf, 13 dpf and 28 dpf.

Wild animals

N/A

Reporting on sex

N/A at the developmental stages used.

Field-collected samples

N/A

Ethics oversight

Zebrafish experimental procedures were carried out in compliance with the ethical guidelines of the German animal welfare law and approved by the local government (Landesuntersuchugsamt Rheinland-Pfalz, Germany – 23 177-07/G20-1-033 and UK Home Office PPL number PEF291C4D).

Note that full information on the approval of the study protocol must also be provided in the manuscript.
